# The Transition from Unfolded to Folded G-Quadruplex DNA Analyzed and Interpreted by Two-Dimensional Infrared Spectroscopy

**DOI:** 10.1021/jacs.3c04044

**Published:** 2023-08-30

**Authors:** A. Larasati Soenarjo, Zhihao Lan, Igor V. Sazanovich, Yee San Chan, Magnus Ringholm, Ajay Jha, David R. Klug

**Affiliations:** † Department of Chemistry, Imperial College London, White City Campus, London W12 0BZ, United Kingdom; ‡ Rosalind Franklin Institute, Harwell, Oxfordshire OX11 0QX, United Kingdom; § Central Laser Facility, Research Complex at Harwell, STFC Rutherford Appleton Laboratory, Harwell, Oxfordshire OX11 0QX, United Kingdom; ∥ Hylleraas Centre for Quantum Molecular Sciences, Department of Chemistry, UiT The Arctic University of Norway, N-9037 Tromsø, Norway; ⊥ Department of Pharmacology, University of Oxford, Oxford, OX1 3QT, United Kingdom

## Abstract

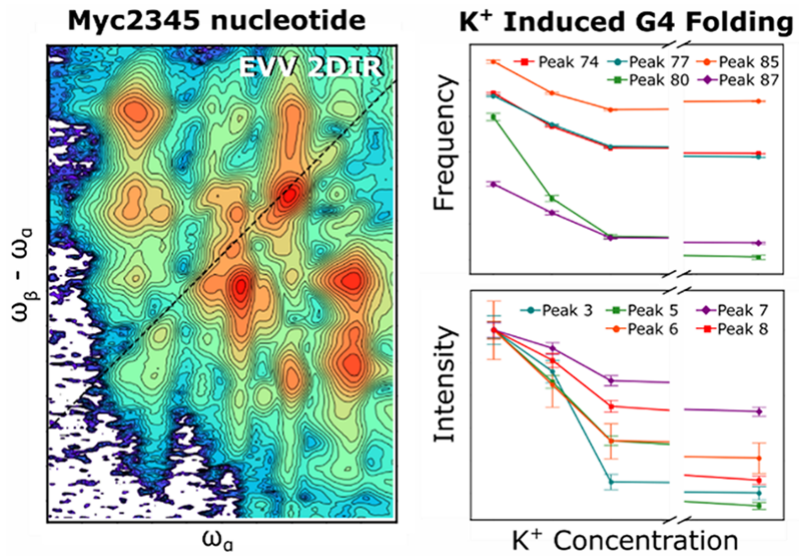

A class of DNA folds/structures known collectively as G-quadruplexes (G4) commonly forms in guanine-rich areas of genomes. G4-DNA is thought to have a functional role in the regulation of gene transcription and telomerase-mediated telomere maintenance and, therefore, is a target for drugs. The details of the molecular interactions that cause stacking of the guanine-tetrads are not well-understood, which limits a rational approach to the drugability of G4 sequences. To explore these interactions, we employed electron-vibration-vibration two-dimensional infrared (EVV 2DIR) spectroscopy to measure extended vibrational coupling spectra for a parallel-stranded G4-DNA formed by the Myc2345 nucleotide sequence. We also tracked the structural changes associated with G4-folding as a function of K^+^-ion concentration. To classify the structural elements that the folding process generates in terms of vibrational coupling characteristics, we used quantum-chemical calculations utilizing density functional theory to predict the coupling spectra associated with given structures, which are compared against the experimental data. Overall, 102 coupling peaks are experimentally identified and followed during the folding process. Several phenomena are noted and associated with formation of the folded form. This includes frequency shifting, changes in cross-peak intensity, and the appearance of new coupling peaks. We used these observations to propose a folding sequence for this particular type of G4 under our experimental conditions. Overall, the combination of experimental 2DIR data and DFT calculations suggests that guanine-quartets may already be present before the addition of K^+^-ions, but that these quartets are unstacked until K^+^-ions are added, at which point the full G4 structure is formed.

## Introduction

G-quadruplexes (G4) are noncanonical DNA secondary structures which can form in guanine-rich DNA sequences.^1,2^ G4s can be unimolecular, bimolecular or tetramolecular, and structures vary in relative strand direction, loop length and composition, and glycosidic bond rotation.^2^ Over 700,000 G4 sequences have been identified in the human genome using next-generation genome sequencing.^3^ Both experimental methods^3^ and computational algorithms^4,5^ suggest a high percentage of G4-forming sequences are found in gene promoter regions and telomeres. The formation of G4 structures in these regions is thought to inhibit the growth of cancer cells by inhibiting telomere maintenance^6,7^ and impeding the transcription of oncogenes.^8,9^ The use of G4-stabilizing ligands is therefore being explored as a potential molecular therapeutic approach to the suppression and elimination of cancerous cells. As such, structural insight into G4s and G4-ligand complexes is useful to assist in ligand design.

**Figure 1:**
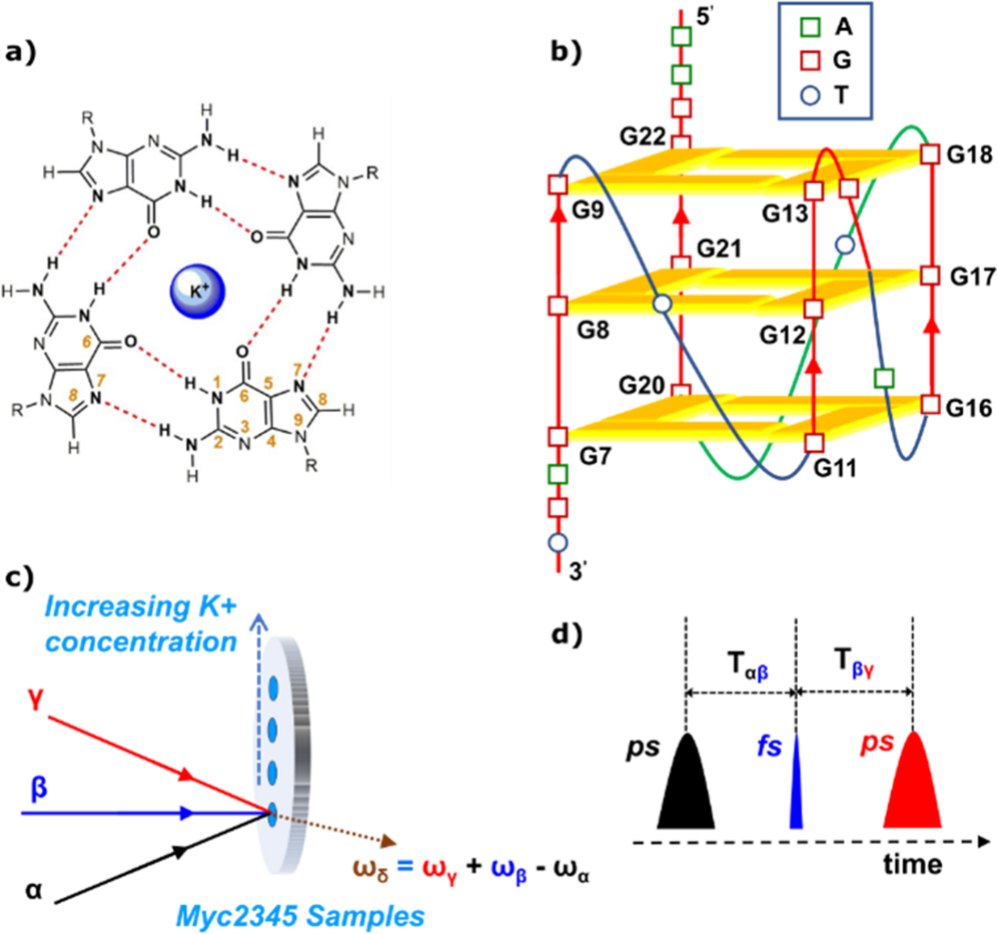
EVV 2DIR spectroscopy as a tool to study G-quadruplexes. (a) Chemical structure of a G-quartet with metal ion coordination to O_6_ oxygen. The structure shows a Hoogsteen hydrogen bonding motif between four guanine bases of a planar G-quartet. (b) Structure of the intramolecular, parallel, propeller-type G-quadruplex formed by Myc2345 in the presence of K^+^. The K^+^ ions lie between two G-quartets (which has not been shown here for better visualization of nucleotide folded structure). Yellow rectangles represent guanine bases in the C2-endo/anti conformation. (c) Laser pulses used in EVV 2DIR experiments are shown schematically. The third *probe beam* (ω_γ_), nonresonant in the electronic domain, is used to look at the polarization produced by the two resonant IR beams, while the two infrared laser beams, ω_α_ and ω_β_ cause resonant vibrations of the system. The signal is anti-Stokes to probe beam photons, ω_δ_ = ω_γ_ + ω_β_ – ω_α_. (d) Pulse sequence showing time delays between different pulses. The time delays used for all experimental measurements described in this work are *T*_αβ_ = *T*_βγ_ = 0.4 ps.

G4s consist of planar G-quartets, formed by four guanine bases hydrogen-bonded in a Hoogsteen motif (Figure 1a), and these quartets can stack vertically to produce the full G4 structure. This stacking requires the presence of metal ions to stabilize the structure. Overall, G4s are therefore stabilized by the Hoogsteen hydrogen bonding to form quartets of bases, and π–π stacking interactions between G-quartets to form the G4 structure. There are also specific metal-ion interactions (direct inner spheres coordination) with the O6 carbonyl oxygen of the guanine bases as well as nonspecific electrostatic ionic interactions. However, the relative importance and details of the combination of these multiple factors to the overall structure is not entirely clear.^10,11^

The structural analysis of nucleic acids has been predominantly approached through X-ray crystallography, NMR spectroscopy, and cryo-EM, each providing complementary information.^12−14^ 2DIR spectroscopy is an optical analogue of 2D NMR spectroscopy that measures the coupling between vibrational modes, which can occur electrically through space and additionally mechanically through bonds. The stretching frequencies of carbonyl groups in DNA bases are known to be sensitive to DNA conformation and conventional 2DIR methods have revealed that it is primarily strong coupling between carbonyl stretches in base pairs and between stacked bases that accounts for the frequency’s observed sensitivity to structure.^15,16^ 2DIR spectroscopy has previously been used to explore DNA structural elements in a number of ways. For example, the analysis of DNA-ligand binding using 2DIR spectroscopy has also led to the development of an induced fit model for the binding of Hoechst33258 to the minor groove.^17^ 2DIR spectroscopy has also been used to study G4 DNA structures.^18,19^ In these studies, the dependence of the guanine carbonyl stretch frequency on the G-tract length was used to determine the preferred number of stacked G-quartets in extended G-tracts.^18^ Polarization-dependent 2DIR spectroscopy was also used to distinguish between parallel and antiparallel G4s.^19^

In this paper, we report the use of a variant of 2DIR spectroscopy called electron-vibration-vibration (EVV), which has the ability to measure across a wide spectral window and recover ∼100 distinct and unique vibrational couplings for a typical protein or nucleic acid sample. EVV 2DIR spectroscopy, also known as DOVE FWM spectroscopy, was first demonstrated by Wright and Cho.^20^ It is a four-wave mixing technique in which two mid-IR pulses excite two vibrational states, e.g., a fundamental vibration and a two-quantum combination state. A third nonresonant pulse using photon energies high enough for single photon counting induces a Raman transition if the two excited modes are coupled, and it is this Raman scattering which is detected as a homodyne signal. Since its inception, the technique has been applied to the geometry determination of complexes,^21^ protein identification,^22^ quantification of tyrosine nitration in peptides,^23^ tissue imaging,^24^ and the detection of protein–ligand complexes.^25^

In this work, we use EVV 2DIR spectroscopy in combination with quantum chemistry calculations based on density functional theory to study the structural changes that occur in Myc2345 due to the addition of potassium ions, which shifts the position of thermodynamic equilibrium from an unfolded to a folded G4. The 22-mer DNA sequence Myc2345 forms an intramolecular, propeller-type, parallel-stranded G4 in the presence of potassium ions.^26^ The high intracellular concentration of K^+^ ions suggests that K^+^-bound structures prevail under physiological conditions, which makes quadruplex conformations with K^+^ of relevance.^27^

## Materials and Methods

### EVV 2DIR Spectroscopy

The setup comprised a dual Ti:sapphire amplifier (Thales Laser), synchronized by a single oscillator seed source (Femto Laser), producing two 800 nm, 10 kHz, 0.8 mJ beams with fwhm pulse durations and bandwidths of 1 ps, 20 cm^–1^ (α) and 50 fs, 300 cm^–1^ (β).^28^ Optical parametric amplifiers (4W pump power, Light Conversion TOPAS) were used to tune the central frequency of the beams: ω_α_ was varied from 1250 to 1750 cm^–1^ in step sizes of 5 cm^–1^, and ω_β_ was set to 3200 cm^–1^. The γ beam was produced by running an arm of the femtosecond beam through an etalon, producing 800 nm, 1 ps pulses with a bandwidth of 12 cm^–1^. All three beams were focused on the sample in a geometry that satisfied phase matching conditions. Time delays between each pulse were created using delay stages and were set to *T*_αβ_ = *T*_βγ_ = 0.4 ps. The FWM signal was detected by a combination of a spectrograph and a charge coupled device camera.

### Sample Preparation

Myc2345 was purchased from Eurogentec and was used without further purification. Myc2345, d(5′-TGAGGGTGGGGAGGGTGGGGAA-3′), was dissolved in a Bis-Tris buffer solution in Nanopure water (10 mM, pH 7.1) to a concentration of 1 mM. KCl was added to the sample at concentrations of 0, 5, 10, and 100 mM. The sample solution was vortexed for 10 s, annealed at 95 °C for 2 min, and cooled to room temperature. The sample solution was formed into gel spots by depositing 1 μL on a glass coverslip and allowing it to dry in a sealed sample cell of 85% relative humidity, created by depositing 6 × 2 μL spots of a saturated aqueous solution of KCl. The sample cell comprised of the glass coverslip, a 1/4 in. thick spacer and a 2 mm thick CaF_2_ disc as the front window. As the spots shrink when they form the gel phase, the final potassium concentration is higher than that of the initial solution.

### Data Processing

#### Glass

A spectrum of the glass coverslip measured at time delays of *T*_αβ_ = *T*_βγ_ = 0 was used to normalize the sample spectra, to account for varying the OPA output energies across the wavelength ranges. The glass spectrum was smoothed by averaging over the nearest neighbors. Interference fringes were observed in the ω_β_–ω_α_ axis of the spectrum. To remove the fringes, linear interpolation was applied along the ω_β_–ω_α_ axis, maintaining the same number of data points. A low-pass brick wall filter was applied to the Fourier transform of the subsequent glass spectrum, using a cutoff frequency of 0.05. The 2DIR spectrum of glass after each step are presented in Figure S7.

#### DNA Sample Spectra

The sample spectra were smoothed by averaging over the nearest neighbors. Linear interpolation was applied along the ω_β_–ω_α_ axis to match the glass spectrum. A background subtraction was applied to the spectra by subtracting the mean intensity in the region ω_α_ = 1325–1375 cm^–1^, ω_β_–ω_α_ = 1190–1210 cm^–1^. The spectra were then background subtracted and normalized on the glass spectrum. As the FWM signal is proportional to the square of the analyte, the intensities of the spectra were square-rooted. The spectra were also normalized at the highest intensity peak at ω_α_ = 1525 cm^–1^, ω_β_–ω_α_ = 1450 cm^–1^. Each sample spectrum is the average of three measurements.

### 2D Gaussian Fitting

To estimate the number of peaks in the experimental 2D spectra, line-cuts at different frequencies were analyzed. This provided a reasonable estimate for the number of Gaussian functions (102) to be used for the 2D Gaussian fitting. Each of the spectra were fitted with a sum of 102 2D Gaussian functions using the nonlinear least-squares fitting function *scipy.optimize.curve_fit* on Python. The Trust Region Reflective algorithm was used to perform the minimization of the Chi-squared value. Errors in the parameters of the fit were propagated from the residuals of the best fit. The 100 mM spectrum was first fitted with no fixed parameters. The initial guess locations were based on visual inspection of line cuts of the spectrum, and these were allowed to vary by ±15 cm^–1^. The initial guess intensities were randomly generated numbers between 0 and 1 and were allowed to vary within the same range. The initial guess widths were 25 cm^–1^ for all peaks in both dimensions, and these were allowed to vary between 5 and 40 cm^–1^. Each of the sample spectra were then fitted using the optimized parameters from the initial 100 mM fit as the initial guesses. All parameters were fixed, apart from intensities, which were allowed to vary from 0 to 1, and the location of peaks that were observed to shift in frequency with respect to potassium ion concentration in the line cuts of the spectra. These shifting peaks were allowed to vary by ±30 cm^–1^ in the dimension the shift is observed.

As we are averaging over the phase envelope of the electric field, the complex susceptibility will only affect EVV 2DIR spectra when spectral features overlap and interfere.^29^ In order to mitigate the effect of this limitation on the interpretation of our data, we restrict most of our assignments to whole rows and whole columns of multiple peaks, where each row and each column can be associated with the coupling of one mode to multiple other modes. While individual features within these rows and columns are modulated by interference, the appearance of the whole row and column is likely less sensitive to this effect.

### EVV 2DIR Spectra Calculations

The computational EVV 2DIR spectra in this work were obtained via a hybrid method based on the Gaussian software^30^ (Gaussian 16, Revision B.01) and in-house scripts. In the Supporting Information to this paper, we include relevant optimized structures and input files for Gaussian.

Following the work of Kwak et al.^31^ and considering only the excitation sequence where, in a normal-mode factorization of the vibrational wave function, the first incident IR pulse induces a one-quantum excitation to a singly excited state and the second IR pulse induces a two-quantum excitation to a doubly excited state in which at least one of the mode indices corresponds to that of the singly excited state, and furthermore assuming temporal disjointness of these two pulses, the relevant contributions to the third-order nonlinear susceptibility χ^(3)^, which is the central quantity for the EVV 2DIR spectral intensities, can by a perturbation-theory development truncated at the first order of anharmonicity in the involved polarization properties (called electrical anharmonicity) and vibrational potential (called mechanical anharmonicity), be expressed as the sum χ^(3)^ = χ_*E*_^(3)^ + χ_*M*_^(3)^ of electrical and mechanical anharmonicity contributions χ_E_^(3)^and χ_M_^(3)^, respectively. In the present work, we have ignored the contribution from χ_M_^(3)^ and considered only χ_E_^(3)^, which we believe will still be sufficient for observations of a qualitative nature. The evaluation of χ_E_^(3)^involves calculating the relevant vibrational energy levels (of singly and doubly excited states) and, under the excitation pattern considered, the normal-mode geometric first- and second-order derivatives  and  of the molecular dipole moment μ and first-order derivatives  of the molecular polarizability, α (*Q*_*i*_ and *Q*_*j*_ refer to normal modes). We refer to ref (31) for more details of these expressions, noting that our computational treatment assumes uniform external field strengths over the spectral region considered, thus not considering any pulse frequency envelope nonuniformity in experiment, and employs Gaussian line-shapes rather than the Lorentzian line-shapes dictated by the ref (31) development.

*In vacuo* G-quartet and G-quadruplex molecular structures were first prepared using PyMol^32^ and then optimized with Gaussian at the density functional theory (DFT) level of theory using the B3LYP functional,^33−37^ and using the 6-31G(d,p) basis set,^38−40^ employing the Int = UltraFine grid granularity parameter and SCF = VeryTight convergence criterion. The optimized G-quartet and G-quadruplex structures were subsequently used with Gaussian, employing the same level of theory and basis set as for the geometry optimization, to calculate harmonic vibrational normal-mode frequencies ω_*i*_ and the dipole moment and polarizability and the first-order normal-mode derivative of the dipole moment, using numerical differentiation with in-house scripts of the dipole moment, dipole moment first derivative, and polarizability calculated at normal-mode displaced geometries to obtain the derivatives described above for the EVV 2DIR spectral intensities. We assumed a harmonic regime for the vibrational energy levels with a uniform scaling parameter of 0.96,^41,42^ thus resulting in no difference between the sum ω_*i*_ + ω_*j*_ of singly excited energy levels and the combination/overtone energy level ω_*i*__+*j*_ involving the same normal mode indices. The resulting data was then processed using in-house scripts into two-dimensional spectral intensities, each peak being dressed with 2D Gaussian line shapes with a line width of 20 cm^–1^ (fwhm), and subsequently rendered using the Matplotlib Python library.^43^

## Results

The details of the EVV 2DIR method, data analysis, and sample preparation are provided in the “Materials and Methods” section.

### Electron-Vibration-Vibration Two-Dimensional Infrared (EVV 2DIR) measurements

**Figure 2:**
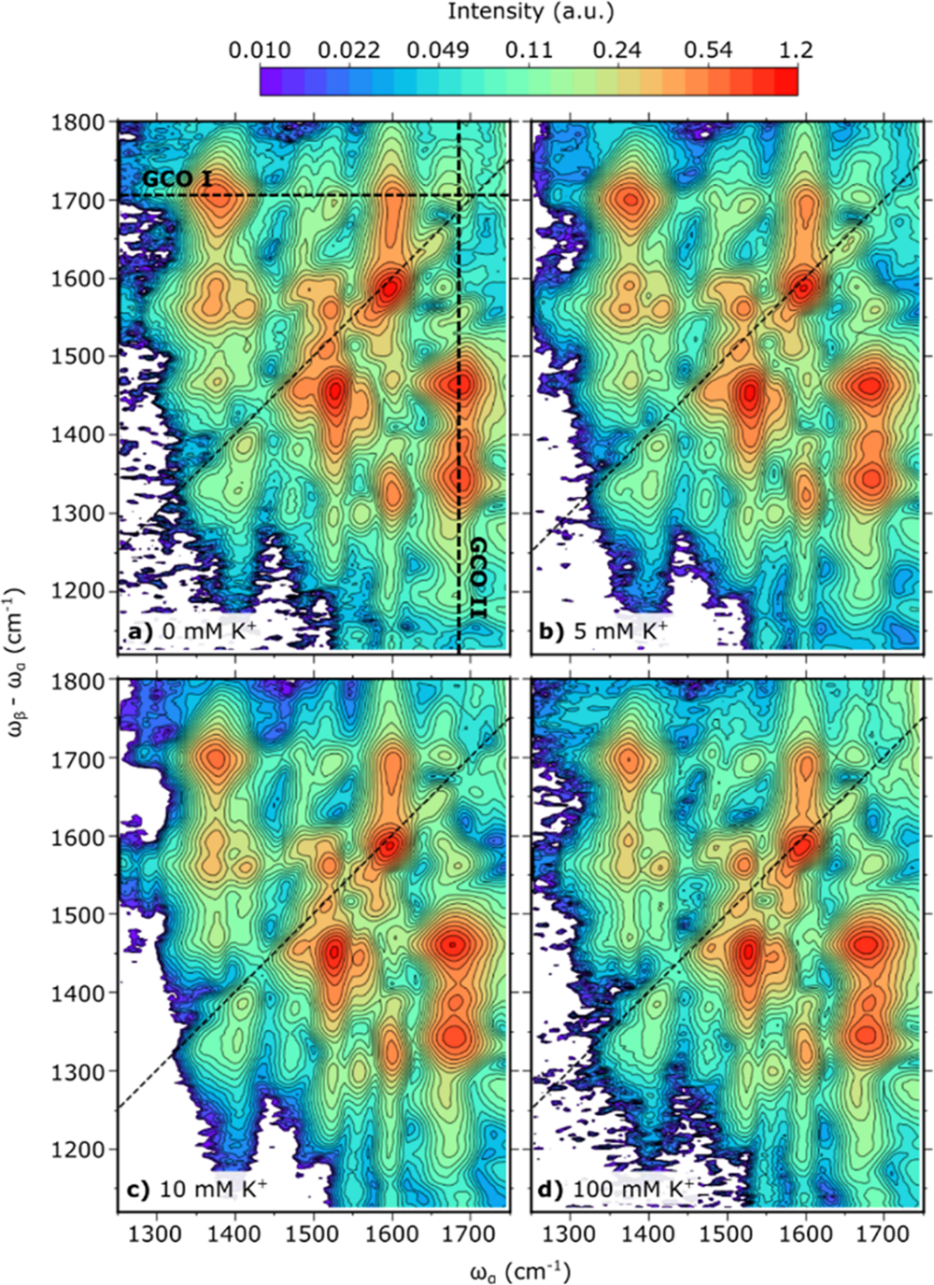
Experimental EVV 2DIR spectra of G4-forming DNA sequence, Myc2345, with varying [Myc2345]:[K^**+**^]. (a–d) Processed EVV 2DIR spectra of 1 mM Myc2345 in the presence of 0 mM (a), 5 mM (b), 10 mM (c), and 100 mM (d) K^+^. All spectra have been normalized with respect to the highest intensity peak at ω_α_ = 1525 cm^–1^, ω_β_–ω_α_ = 1450 cm^–1^. In EVV 2DIR spectroscopy, when considering the situation where combination bands and overtones are excited by one IR pulse at frequency ω_β_ while a one-quantum transition is excited by a second IR pulse at frequency ω_α_, the coupling of one quantum transitions to their overtones is expected to fall on the dotted “overtone diagonal” line, although due to other factors such as anharmonicity, they do sometimes fall slightly below it.^44^ Off-diagonal features arise from the coupling of vibrational modes at frequencies ω_α_ and ω_β_–ω_α_, with ω_β_ exciting combination bands with frequencies that are approximately the sum of the two coupled modes. Horizontal and vertical dashed lines in (a) highlight the row and column of cross-peaks with frequencies that are dependent on potassium ion concentration, which have been assigned to the coupling of a guanine carbonyl stretch mode with various other modes. The guanine carbonyl stretch mode giving rise to the row of cross-peaks (GCO I) is higher in frequency than the guanine carbonyl stretch mode giving rise to the column of cross-peaks (GCO II).

The experimental 2DIR spectra of 1 mM Myc2345 in the presence of 0, 5, 10, and 100 mM K^+^ are presented in Figure 2. In EVV 2DIR spectroscopy, one infrared pulse excites one vibrational band, while the other excites another. In this work, we consider only the situation where, in a normal-mode factorization of the vibrational wave function, the first pulse induces a one-quantum excitation to a singly excited state, and the second induces a two-quantum excitation to a doubly excited state in which at least one of the mode indices corresponds to that of the singly excited state. The coupling is read out by a visible pulse which Raman-scatters from the coherence if the two excited bands are coupled and does not scatter if they are not. The measured four-wave mixing signals are plotted with the Raman excitation frequency *ω*_*γ*_ subtracted, yielding a difference frequency axis ω_β_–ω_α_ (*Y*-axis) as a function of ω_α_ (*X*-axis). As ω_β_ excites a combination band, the two axes approximately correspond to the frequencies of the two fundamental vibrations that contribute to the combination band. This leads to rows and columns of features being observable in the data. These correspond to a particular vibrational mode, coupling to multiple other modes. Because the coupling density is high, multiple coupling peaks overlap in these spectra which means that peak positions do not necessarily represent the positions of couplings but instead are sometimes the peaks caused by the presence of multiple couplings.

**Figure 3:**
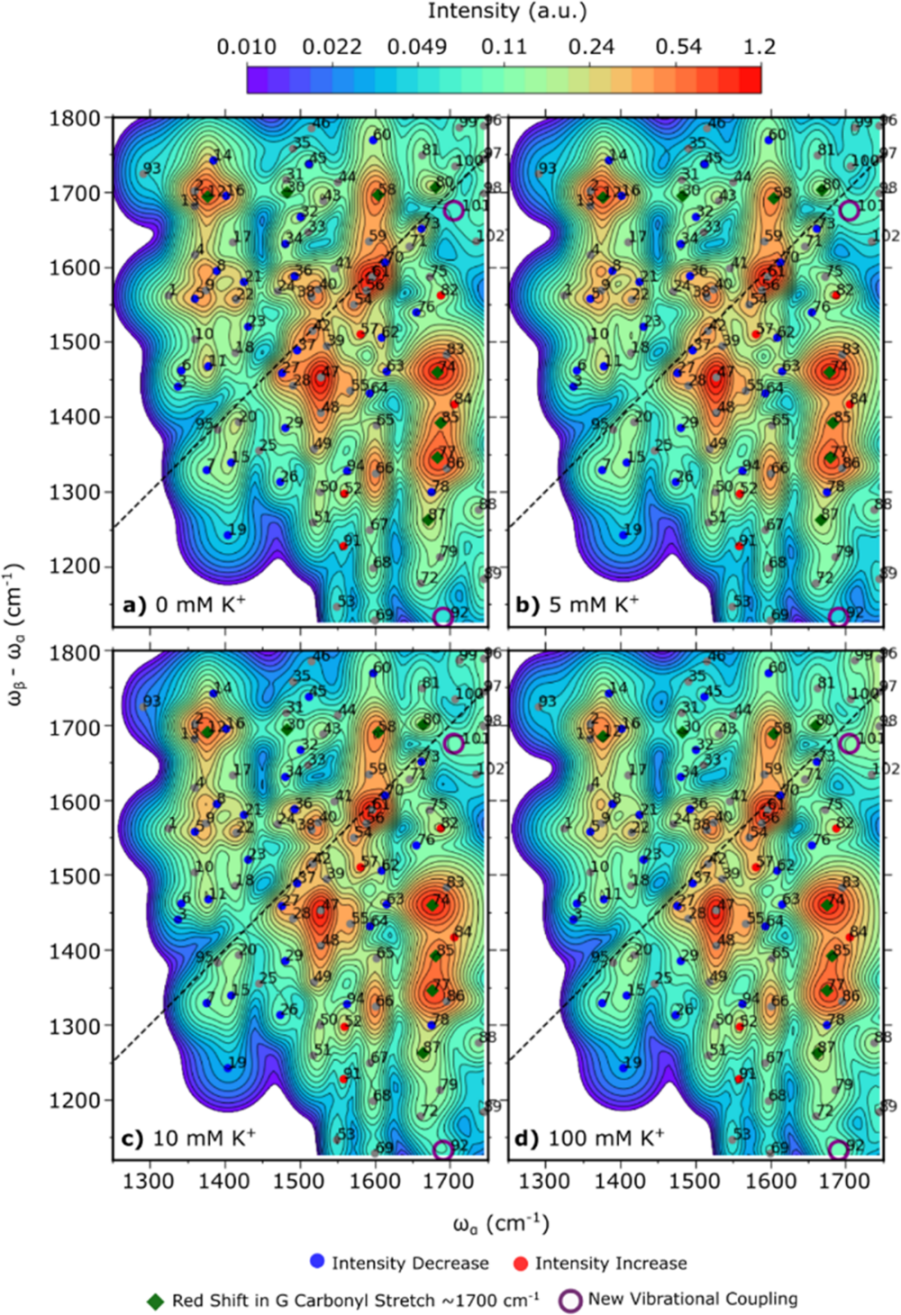
Fitted EVV 2DIR spectra of G4-forming DNA sequence, Myc2345, with varying [Myc2345]:[K^**+**^]. (a–d) Processed EVV 2DIR spectra of 1 mM Myc2345 in the presence of 0 mM (a), 5 mM (b), 10 mM (c), and 100 mM (d) K^+^ fitted with a sum of 102 Gaussian functions. Markers denote the central frequencies of the 102 Gaussian functions used in the fitting of the respective spectra. The diagonal dotted line shows the overtone diagonal. Features along the diagonal arise from the coupling of a fundamental mode at ω_α_ and its overtone at ω_β_. Off-diagonal features arise from the coupling of vibrational modes at frequencies ω_α_ and ω_β_–ω_α_. Spectral changes observed as a result of K^+^-induced structural changes in the DNA include a decrease in cross-peak intensity (blue circles); an increase in cross-peak intensity (red circles); the red-shifting of guanine carbonyl stretch frequencies at ∼1700 cm^–1^ (green diamonds); and the appearance of new vibrational couplings (purple circles).

**Figure 4:**
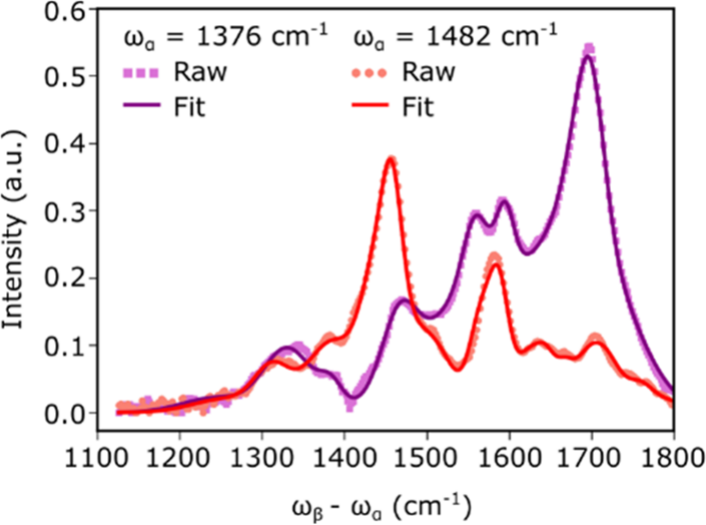
Line cut comparison of raw and fitted EVV 2DIR spectra of Myc2345. Raw (dotted line) and fitted (solid line) EVV 2DIR spectra of 1 mM Myc2345 in the presence of 100 mM K^+^ at ω_α_ = 1376 cm^–1^ (purple) and ω_α_ = 1482 cm^–1^. A comparison of raw and fitted data at other ω_α_ and ω_β_–ω_α_ frequencies can be found in Figure S1.

In order to disentangle these complex spectra as much as possible, the 2DIR spectra are fitted with two-dimensional Gaussian functions, which provide a good approximation to the shape of the typical EVV 2DIR coupling feature. In this case, a minimum of 102 2D Gaussian functions is required, revealing the presence of at least 102 distinct couplings. The fitted 2DIR spectra are presented in Figure 3, with superimposed markers denoting cross-peak central frequencies from the fit. As expected, these do not always perfectly coincide with the peaks of the raw unfitted data. Vertical line-cuts comparing raw and fitted spectra are presented in Figure 4 which demonstrates the high signal-to-noise ratio of this data set and the goodness of the fit. Comparisons of raw and fitted data at other frequencies can be found in Figure S1 along with Chi-squared values in Table S1. In this section we now focus on the frequency shifts, intensity changes, and new vibrational couplings that occur as a result of structural changes on K^+^ ion addition.

**Figure 5:**
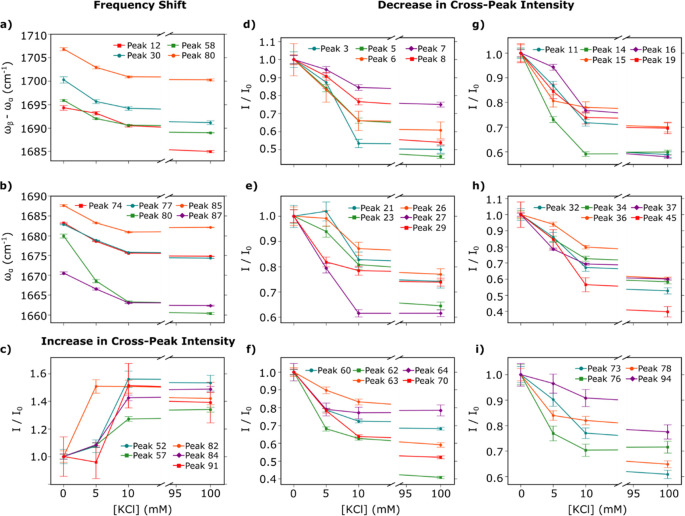
Frequency shifts and intensity changes observed in cross-peaks as a result of K^**+**^-induced formation of full G4 structure. (a,b) Frequency-potassium ion concentration dependence of the guanine carbonyl stretch mode, GCO I (a) and GCO II (b). (c–i) Relative intensity of peaks exhibiting an increase (c) and a decrease (d–i) in cross-peak intensity as a function of potassium ion concentration. Intensity is presented as a fraction of the intensity in the absence of K^+^ ions. Peaks presented here show an overall intensity difference of ≥20%. Intensity-ion concentration dependence of the remaining peaks is presented in Figure S2.

It is observed that multiple peaks in the region of in-plane base vibrations at around ∼1700 cm^–1^ red-shift with increasing K^+^ concentration, as shown in Figures 5a,b. Peaks 12, 30, and 58 red-shift along the ω_β_–ω_α_ axis; peaks 74, 77, 85, and 87 red-shift along the ω_α_ axis; and peak 80 red-shifts along both axes. The observed red shifts are consistent with the weakening of the guanine carbonyl bonds as Hoogsteen hydrogen bond formation and K^+^ coordination occurs. Interestingly, the ω_β_–ω_α_ frequency representing guanine carbonyl stretching appears at a frequency higher than the ω_α_ frequency. In the absence of potassium ions, the ω_β_–ω_α_ frequency associated with guanine carbonyl stretching is in the range 1694–1707 cm^–1^, whereas the ω_α_ guanine carbonyl stretching frequency is observed between 1671 and 1688 cm^–1^. Interpretation of this highly unusual observation can be found in the “Discussion” section. Additionally, the guanine carbonyl stretching frequencies observed in the absence of potassium ions is higher than the single strand guanine frequency typically observed between 1660 and 1673 cm^–1^,^45^ and this observation hints at the possibility of some pre-existing structure in the absence of cations.

It is well-known that nucleic acids exhibit changes in absorbance (hypochromism and hyperchromism) in the UV/vis region of the spectrum, which is thought to be caused by base stacking interactions.^46−48^ Hypochromism and hyperchromism are reflected in the Raman line intensities of ring modes, which has previously been used to monitor conformational changes in nucleic acids.^49−55^ In the EVV 2DIR spectra, we focus on peaks that exhibit a monotonic increase/decrease in intensity and those that also show a minimum intensity change of 20% between the 0 K^+^ and 100 mM K^+^ spectra. We identify 29 peaks that decrease in intensity as K^+^ concentration increases (Figure 5d–i). The majority of these peaks appear at frequencies characteristic of guanine ring vibrations (1476–1495 cm^–1^, 1564–1568 cm^–1^, and 1575–1590 cm^–1^).^45^ Additionally, five peaks have been observed to increase in intensity as the K^+^ concentration increases (Figure 5c). Assignment of these features and a description of the mechanisms for coupling increases and decreases are given in the discussion. The intensity traces of the remaining peaks as a function of K^+^ ion concentration are shown in Figure S2.

**Figure 6:**
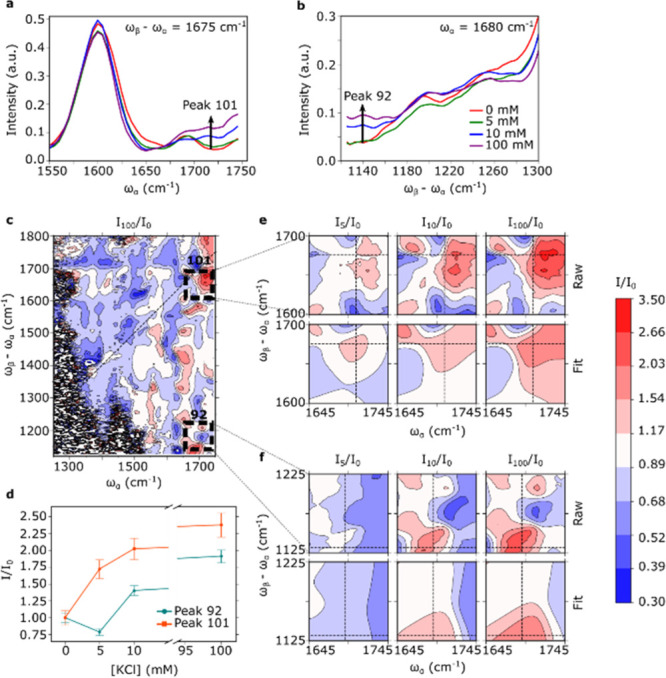
New coupling peaks 92 (ω_α_= 1691 cm^–1^, ωβ–ω_α_= 1132 cm^–1^) and 101 (ω_α_= 1705 cm^–1^, ω_β_–ω_α_= 1675 cm^–1^) appear due to K^**+**^-induced G4 formation. (a,b) Line cuts of experimental EVV 2DIR spectra of 1 mM Myc2345 with 0, 5, 10, and 100 mM K^+^ showing new peaks 101 (a) and 92 (b) appearing as [K^+^] is increased. (c) Experimental spectrum of 1 mM Myc2345 in the presence of 100 mM K^+^ as a fraction of the spectrum of Myc2345 in the absence of ions. Dashed black line boxes show regions where new coupling peaks 92 (bottom) and 101 (top) appear. (d) Relative intensity of new peaks 92 and 101 as a function of [K^+^]. Intensity is presented as a fraction of the intensity in the absence of K^+^ ions. (e) Raw (top) and fitted (bottom) spectra of 1 mM Myc2345 with 5 (left), 10 (center), and 100 mM K^+^ (right), as a fraction of the spectrum of 1 mM Myc2345 in the absence of ions, showing the gradual appearance of peak 101 as [K^+^] is increased. (f) Raw (top) and fitted (bottom) spectra of 1 mM Myc2345 with 5 (left), 10 (center) and 100 mM K^+^ (right), as a fraction of the spectrum of 1 mM Myc2345 in the absence of ions, showing the gradual appearance of peak 92 as [K^+^] is increased. Vertical and horizontal dashed lines show the peak positions according to the 2D Gaussian fit.

The folding of G4 from a relatively disordered to a relatively ordered structure would be expected to produce a few new coupling peaks as chemical groups previously separated are brought closer together. Figure 6a,b display line cuts of experimental EVV 2DIR spectra showing new peaks 101 (ω_α_ = 1705 cm^–1^, ω_α_–ω_α_ = 1675 cm^–1^) and 92 (ω_α_ = 1691 cm^–1^, ω_α_–ω_α_ = 1132 cm^–1^), respectively. Fitting of the line cuts is presented in Figure S8 and S9, which show clear evolution of new coupling peaks as [K^+^] concentration is increased. Figure 6e,f present the raw and fitted 2DIR spectra in the region of the observed new peaks 101 and 92, respectively (see Figure S10 for line cuts from the fitting of the entire 2D spectra). Figure 6d presents the intensity–ion concentration dependence of both peaks. New vibrational modes extending over multiple G-quartets, and therefore having greater levels of delocalization, can also result in the appearance of new peaks. These peaks may serve as markers of the G4 formation. Assignments of these peaks will be discussed in the “Discussion” section.

## Discussion

**Figure 7:**
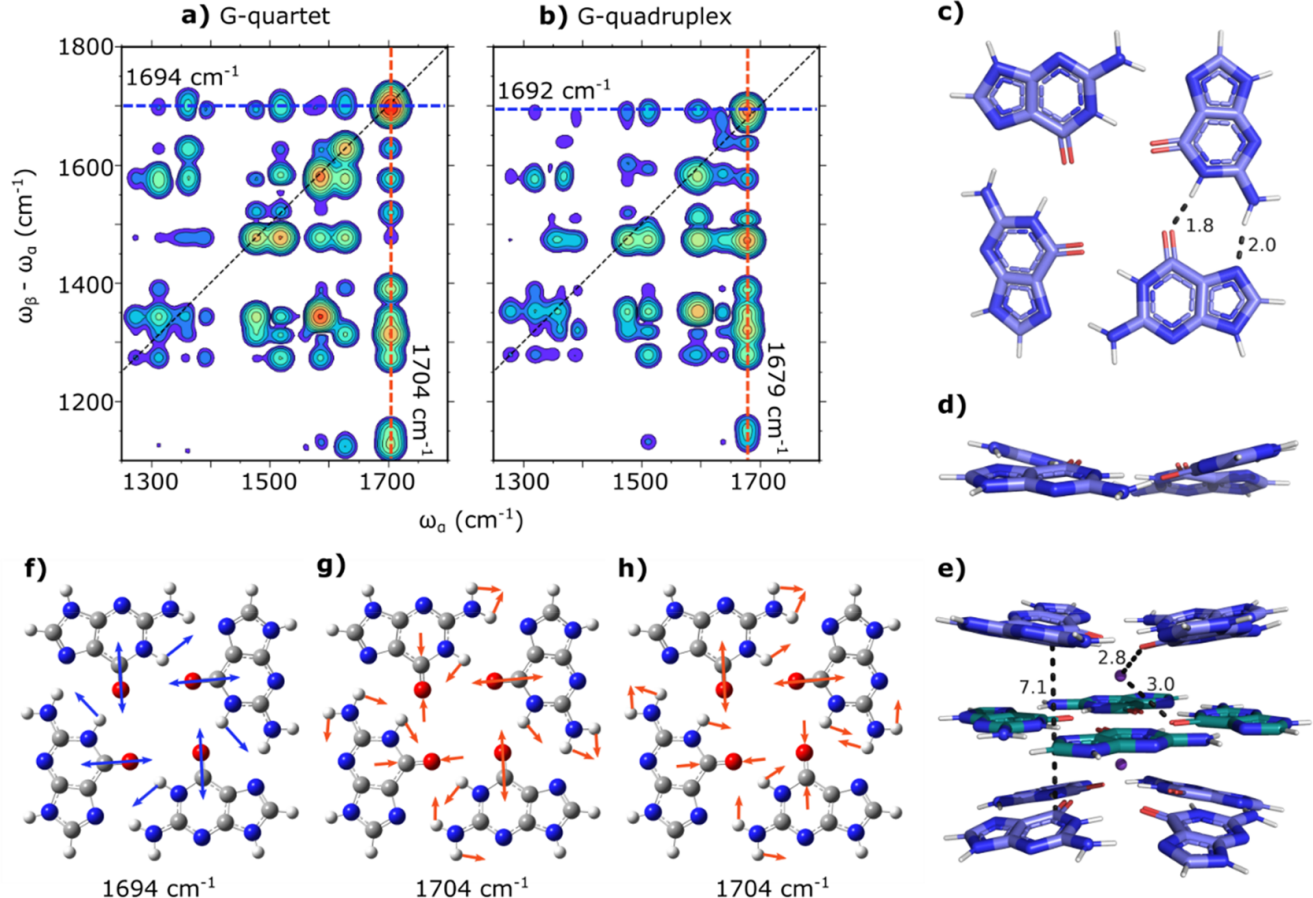
Computational EVV 2DIR spectra of a G-quartet and a G-quadruplex. (a) Computational EVV 2DIR spectrum of a nonplanar G-quartet. Four combinations of guanine carbonyl stretch modes at ∼1700 cm^–1^ exist in a G-quartet. The guanine carbonyl stretch mode giving rise to the highest intensity couplings along the row of cross-peaks is the in-phase guanine carbonyl stretch mode at 1694 cm^–1^ as depicted in (f). Two degenerate out-of-phase guanine carbonyl stretch modes at 1704 cm^–1^, as depicted in (g) and (h), give rise to the highest intensity couplings along the column of cross-peaks. (b) Computational EVV 2DIR spectrum of a G-quadruplex. There are 12 combinations of guanine carbonyl stretches in a G4. The guanine carbonyl stretch mode giving rise to the highest intensity couplings along the row of cross-peaks at 1692 cm^–1^ involves the same motions as the 1694 cm^–1^ G-quartet mode, as seen in (f), across all three quartets of the G4. Similarly, two degenerate guanine carbonyl stretch modes give rise to the highest intensity couplings along the column of cross-peaks at 1679 cm^–1^. They involve the same motions as the 1704 cm^–1^ G-quartet modes, as shown in (g) and (h), but across all three quartets in the G4. Animations of the G4 vibrational modes at 1692 and 1679 cm^–1^ can be found in the Supporting Information. (c,d) Top-down view (c) and side view (d) of the G-quartet structure used to calculate the EVV 2DIR spectrum in (a). (e) Structure of G4 used to calculate the EVV 2DIR spectrum in (b). (f) In-phase guanine carbonyl stretch mode of a G-quartet at 1694 cm^–1^. (g,h) Two degenerate out-of-phase guanine carbonyl stretch modes of a G-quartet at 1704 cm^–1^.

One of the initially surprising aspects of the experimental data is that relatively few completely new couplings are created by the folding of the G4. Although behavior like this may be observed in our calculated spectra, such as in Figure 7a,b, it is useful to reflect on the reasons for this. Chemical moieties such as DNA bases contain many chemical “groups” which couple to each other in a diverse set of ways. The formation of a quartet of bases coupled by Hoogsteen hydrogen bonding might therefore primarily be characterized by the reorganization/recombination of the vibrational states of the individual bases (and the couplings between them) rather than the emergence of altogether new couplings not characterizable in this way. This is loosely analogous to the situation in molecular exciton coupling, where electronic states are reorganized by the new couplings, rather than the number of electronic states being increased.

One of the models for the formation of G4 is the transition between two states: a single strand in the absence of ions and a fully folded G4 in the presence of an excess concentration of potassium ions. However, the frequencies associated with guanine carbonyl stretching in the EVV 2DIR spectrum of Myc2345 in the absence of ions are not characteristic of that of guanine in a single strand.^45^ Nonetheless, the formation of a higher order structure in response to the addition of potassium ions is indicated by a number of observations, namely, the red-shift of the guanine carbonyl stretch frequencies, the observed changes in cross-peak intensities, and the appearance of two new couplings. To gain a better understanding of the structure observed in the absence of ions and the observed spectral changes upon increasing ion concentration, we calculated EVV 2DIR spectra for G4 and its possible precursors. The details of the calculation methodology are provided in the “Materials and Methods” section. We note here that we have in this work not included the so-called mechanical anharmonicity contribution to the expressions for the spectral intensities,^31^ and the calculated spectra are therefore only based on the electrical anharmonicity contribution, here involving second-order derivatives of the molecular dipole moment. We furthermore note that we have employed a uniform scaling parameter for the vibrational energy levels, which were calculated by harmonic vibrational potential approximation, and thus not considered any difference between overtone/combination band energy levels and the corresponding sum of one-quantum energy levels. Finally, we remark that the calculations have been carried out *in vacuo*, not involving any implicit or explicit solvation while the experimental sample existed in the presence of abundant water. Altogether, the level of confidence in the findings associated with the analysis of the quantum-chemical calculations in this work should therefore be considered with these limitations in mind. The overall strategy in this work is to identify and distinguish three possible structure types. These are isolated guanines that are not part of a quartet; guanines arranged in a quartet and guanine quartets stacked to form a G-quadruplex.

### The Initial DNA Structure Contains Guanines in a Structure Resembling a G-Quartet in the Absence of Potassium Ions

Multiple pieces of evidence point toward the presence of pre-existing DNA structure in the absence of ions. The first evidence comes from the observation of the frequency difference between the row and corresponding column of peaks arising from the coupling of a guanine carbonyl stretch mode with various other vibrational modes (Figure 2a). In general, for EVV 2DIR spectroscopy, the rows and columns of cross-peaks relating to the same mode have an approximate reflective symmetry about the overtone line (ω_α_ = ω_β_ – ω_α_). However, this symmetry is frequently broken by a negative frequency shift along the ω_β_–ω_α_ axis. This is because a combination band is usually shifted to a frequency lower than the sum of the fundamental frequencies by the process of mode-coupling. In our experimental spectrum (Figure 2a), the row of cross-peaks with frequencies which depend on potassium ion concentration are assigned to the guanine carbonyl stretch with a frequency between 1694 and 1707 cm^–1^. This frequency is unusually higher than the corresponding column of peaks with frequencies that are also dependent on potassium ion concentration and appears between 1671 and 1688 cm^–1^. Assuming that these are not combination bands that are shifted to a higher frequency than the sum of the constituent fundamentals, this indicates that the guanine carbonyl stretch mode giving rise to the row, which we refer to as GCO I, is not the same one that gives rise to the column, which we refer to as GCO II.

An occurrence of two different guanine carbonyl modes giving rise to what is normally a corresponding row and column of cross-peaks is impossible to reconcile with the spectroscopy of an isolated guanine base (Figure S3). The calculated EVV 2DIR spectra and the associated vibrational analysis do, however, exhibit a comparable behavior for the structures involving coupled guanine bases as in a G-quartet (Figure 7a) or a G4 (Figure 7b). The reason for this is that the roughly symmetric G-quartet structure gives rise to multiple combinations of guanine stretch modes in a manner analogous to simple exciton coupling. Although many different guanine carbonyl centric modes contribute to the column of cross-peaks, 4 in the case of the G-quartet and 12 in the case of the G4 depicted in Figure 7e, the coupling strength of the cross-peaks associated with each carbonyl mode differs, and one mode has much stronger couplings than the others. Interestingly, the dominating carbonyl stretch mode in the row of cross-peaks, is not the same mode that dominates the corresponding column of cross-peaks. Thus, in these calculated spectra, the rows and columns actually originate from different combinations of guanine motions. While the relative difference between the central frequency of the dominating row and column are different and opposite in sign between the experimental and our calculated spectra, this observation can be taken as support for the notion that the observation of differing guanine stretch modes in the rows and columns of the EVV 2DIR spectrum of Myc2345 in the absence of ions, can be due to the presence of a pre-existing structure involving coupled guanine bases. In effect, EVV 2DIR picks out differences in oscillator strength between the one-quantum states of the G-quartet, with one being excited first in the row and another in the corresponding column. As the “excitonic” interactions of the original isolated guanine motions can result in one quantum transitions of different oscillator strength when they combine “excitonically”, then one can have the situation where one state dominates the column while another dominates the row.

Further evidence for the presence of a pre-existing structure in the absence of ions comes from the absolute frequencies of the guanine carbonyl stretches. The frequency of nitrogenous base carbonyl stretches is sensitive to DNA conformation and is often used to determine DNA secondary structure.^45^ The guanine carbonyl stretching frequency is observed at ω_α_ = 1671–1688 cm^–1^ (GCO II), which is higher than a single strand guanine typically observed between 1660 and 1673 cm^–1^,^45^ which could suggest the presence of a pre-existing DNA structure. In fact, circular dichroism measurements of MycL1, a similar G4-forming sequence to Myc2345 (see Figure S4 for sequence comparison), also suggest that some structure can be present in a G4 DNA sequence, even in the absence of potassium ions.^54^

Given the above indications that some structure resembling a G-quartet is present, even in the absence of potassium ions, the obvious question is whether the entire G4 structure may be preformed. Evidence for the absence of a fully stacked G4 complex in the absence of potassium ions comes from four observations. First, in the geometry optimization that we did, a stacked quartet without potassium ions was not found to be energetically stable. This structure is unfavorable due to the repulsive forces between the 12 carbonyl oxygens. As this is a purely enthalpic effect and as entropic forces will only drive the structure apart in a real solvated system, we, therefore, consider the presence of a G4-like structure in the absence of potassium ions to be unlikely.^11^ The three other pieces of evidence for the absence of stacked G-quartets (the entire G4 structure) in the absence of potassium ions are the red-shifts of particular frequencies observed when the potassium ions are added, the intensity changes for multiple peaks upon the addition of potassium, and the formation of new spectral features already known to be associated only with fully stacked G4. We proceed to discuss these in more detail below.

### The Red-Shift of Guanine Carbonyl Stretches Indicate Ion Coordination

G4 formation has previously been monitored by noting a frequency shift in the guanine carbonyl stretch frequency.^56^ Experimentally, we observe a red shift in the frequency of both guanine carbonyl stretch modes, GCO I (Figure 5a) and GCO II (Figure 5b), upon addition of potassium ions. Peak 80, which is a coupling between GCO I and GCO II, shifts diagonally which supports these assignments. According to the existing literature, however, the folding of a single strand to a G4 induces a frequency shift of the guanine carbonyl stretch in the opposite direction.^18,19,56^ The red-shift of the guanine carbonyl frequencies is an interesting observation. There are many possible reasons that can contribute toward our observation. In order to understand our experimental observations, we measured the IR spectrum of the Myc2345 in solution at different K^+^ ion concentrations, which is presented in Figure S11. We observe a similar red shift to that observed in EVV 2DIR measurements. Thus, we can conclude that detection of EVV 2DIR spectra via Raman scattering does not result in the observed anomalous frequency shift and the red-shift of guanine carbonyl frequencies is specific to our sequence. In order to further reconcile the observed discrepancy in guanine carbonyl frequency changes, we compare calculated EVV 2DIR spectra of the proposed initial structure of a G-quartet (Figure 7a) and a G4 (Figure 7b) to experimental spectra of Myc2345 in the absence and presence of potassium ions.

The experimentally determined red-shifts in the guanine carbonyl stretch frequencies caused by the addition of potassium ions are shown in Figures 5a,b. The red-shift in the GCO I and GCO II frequencies is approximately reproduced in the calculated difference spectra between a G-quartet (Figure 7a) and a G4 (Figure 7b). Experimentally, the average frequency shifts in GCO I and GCO II are −8 and −10 cm^–1^, while the calculated frequency shifts are −2 and −25 cm^–1^, respectively. Therefore, our calculations, keeping in mind that they are based on harmonic vibrational wave functions, qualitatively agree with experimental observations and suggest that potassium ion complexation causes a red shift overall. The mechanism of the red-shift of the guanine carbonyl stretch frequencies can be attributed to the ion coordination to the carbonyl oxygen and the stacking of G-quartets. The ion coordination results in the lengthening of the carbonyl bonds from 1.23 to 1.25 Å. Moreover, the resultant stacking of G-quartets results in the delocalization of the guanine carbonyl modes found in the G-quartet at 1704 cm^–1^ (Figure 7g,h) across all three quartets in the equivalent mode in the G4 found at 1679 cm^–1^.

### Changes in Cross-Peak Intensity Indicate Preformed G-Quartet without Full G4 Structure

**Figure 8:**
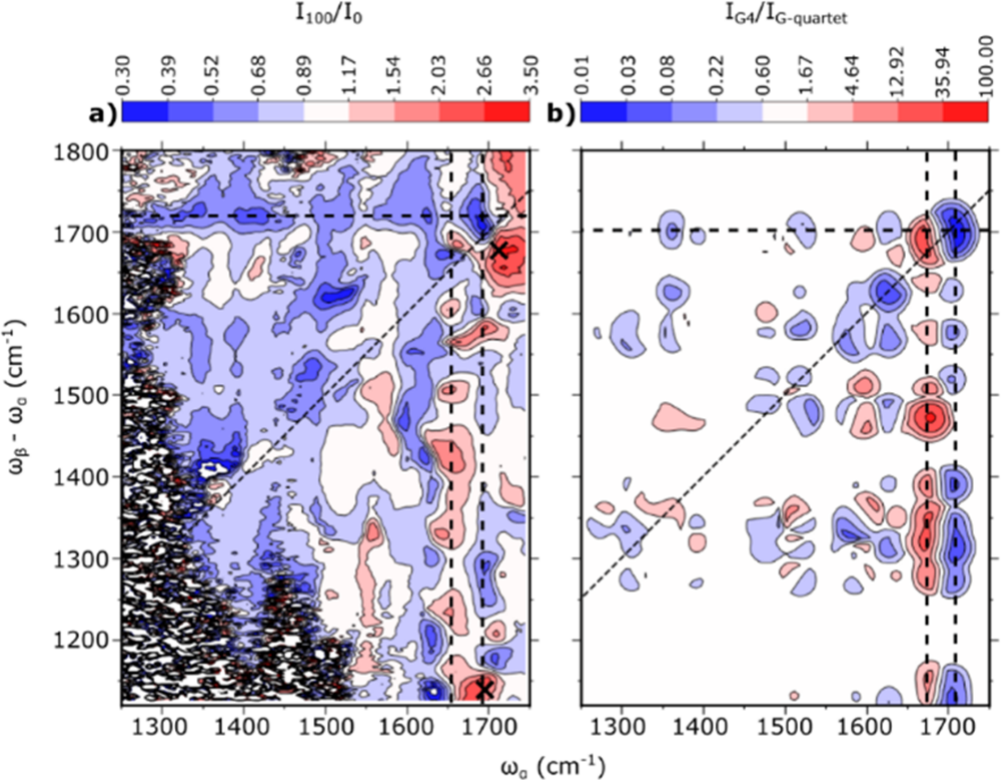
Computational and experimental EVV 2DIR ratio spectra (G-quadruplex/G-quartet). (a) Experimental EVV 2DIR ratio spectra of 1 mM Myc2345 in the presence of 100 mM K+ divided by 1 mM Myc2345 in the absence of ions. (b) Computational EVV 2DIR ratio spectrum of G-quadruplex/G-quartet. The calculated spectra include only electrical coupling, but a similar overall change is observed when mechanical coupling is included. Alternating increases and decreases in coupling observed around ∼1700 cm^–1^ (dashed lines) are due to the red-shifts of guanine carbonyl stretch frequencies. Positive features in the experimental difference spectrum at ω_α_ = 1691 cm^–1^, ω_α_–ω_α_ = 1132 cm^–1^ and ω_α_ = 1705 cm^–1^, ω_α_–ω_α_ = 1675 cm^–1^ (black crosses) are due to the appearance of new peaks 92 and 101, respectively. The remaining difference features are due to intensity changes in cross-peaks as a result of structural changes in the DNA as the potassium ion is introduced. The experimental ratio spectrum is consistent with structural differences between a G-quartet and a G4. Alternating columns of red and blue features on the right-hand side of both spectra is a qualitative indication that the essential features associated with those columns are reflected in both calculation and experiment, as is the absence of that pattern in the corresponding rows.

We find experimentally that a large number of cross-peaks decrease in intensity upon the addition of potassium ions. This observation can also be used to test structural models for the folding, as these reductions in intensities would be manifested in calculations. The measured ratio spectrum caused by the addition of potassium ions and the calculated G-quadruplex/G-quartet ratio spectrum are shown in Figure 8 and have comparable features. Excluding the difference peaks due to frequency shifts and new couplings, both spectra show a majority of negative differences, with some positive difference peaks near or below ω_α_ = 1600 cm^–1^. This overall appearance of reduction in cross-peak intensities, although involving a comparison to calculated spectra based only on electrical anharmonicity, is thus consistent with the G-quartet to G-quadruplex model.

The changes in cross-peak intensity observed going from the G-quartet (Figure 7c,d) to G4 (Figure 7e) can be explained by the change in relative orientation of the guanine bases of a quartet. In the G-quartet, molecular planes of adjacent guanine bases intersect at an angle of 21°. In the G4, this angle is altered to 19° in the terminal quartets and 0° in the central, planar quartet (Figure S6). These structural changes result in changes in the orientation of transition dipole moments, which account for the changes in intensity observed in cross-peaks across the spectrum.

### Peaks 92 and 101 are Markers of a Fully Folded G4 in Myc2345

**Figure 9:**
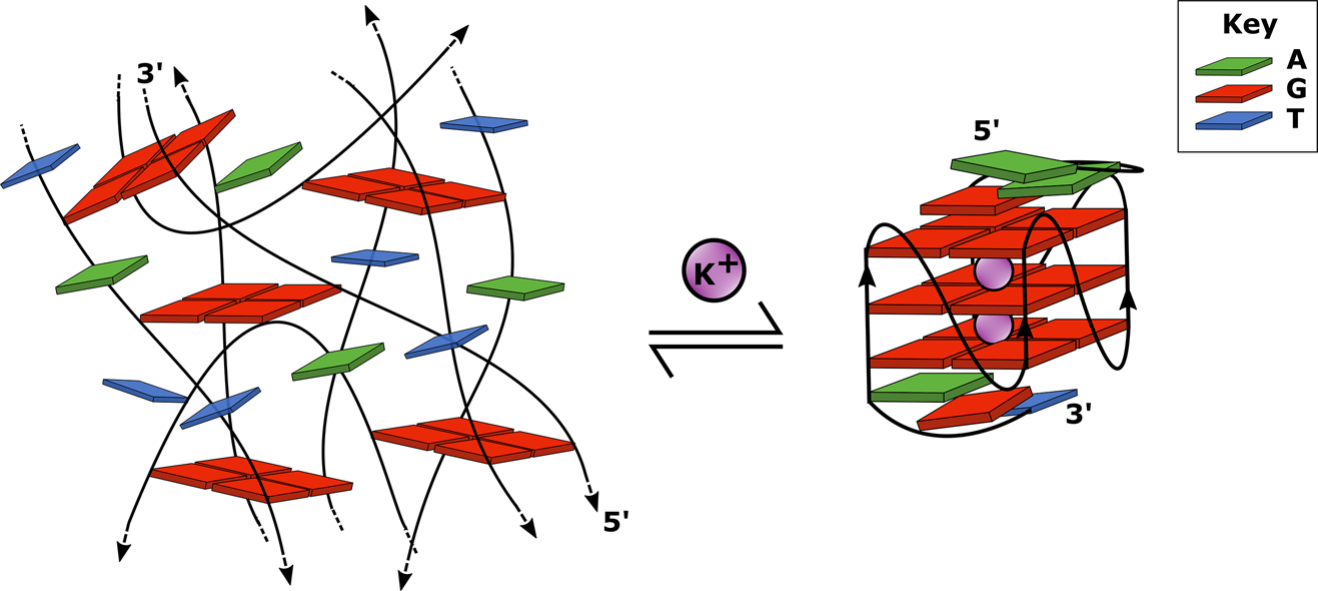
Schematic of the proposed equilibrium between an unstacked G-quartet formed in Myc2345 in the absence of potassium ions and a G4 formed in the presence of potassium ions. Left: A depiction of a possible way in which Myc2345 in the absence of potassium ions might form unstacked G-quartets. Right: Schematic of the G4 structure of Myc2345 in the presence of potassium ions. For clarity, only guanine bases involved in the quartets and bases in the end-caps are included. New coupling peaks 92 and 101 suggest that G/T in the end-caps couple to the core G4 structure.

New cross peaks, which appear as the potassium ion concentration is increased, are potential markers of a fully folded G4. We have identified two new peaks formed on potassium addition: Peak 92 (ω_α_ = 1691 cm^–1^, ω_β_–ω_α_ = 1132 cm^–1^) and Peak 101 (ω_α_ = 1705 cm^–1^, ω_β_–ω_α_ = 1675 cm^–1^). Neither of the peaks appears in the calculated G4 spectrum, which suggests that both peaks could involve vibrational modes outside of the core G4 structural elements included in the calculation (Figure 7e). Using the calculated vibrational frequencies of the G4 structure (Figure 7e), the ω_β_–ω_α_ frequencies of both peaks have been tentatively assigned to vibrations of the core G4 structure: Namely, guanine ring deformations of terminal quartets in peak 92 and NH bends and C=O stretches of all quartets in peak 101. Animations of these G4 vibrations at 1132 cm^–1^ (peak 92) and 1675 cm^–1^ (peak 101) can be found in the Supporting Information. The ω_α_ frequency of both peaks is characteristic of a G C_6_=O_6_ stretch or a T C_2_=O_2_ stretch, which could be located within G/T in the end-caps of Myc2345 (Figure 9) and was not included in the calculation. It is already proposed by other work that bases in the end-caps of G4s interact with the core G4 structure, further stabilizing it. The NMR structure of Myc2345 (Protein Data Bank: 7KBV) is consistent with this idea.^57^

### Summary of the Comparisons between Measured and Computed Spectra

Figure 8b shows the calculated ratio spectrum for G4/G-quartet. This is compared with the experimentally measured ratio spectrum induced by the addition of 100 mM potassium ions (Figure 8a). Qualitatively, a number of key elements stand out as being present in both calculated and measured spectra. First, there is the pattern of a negative column at ∼1600 cm^–1^ next to a lower-frequency positive column. Second there is the absence of such a pattern in the corresponding rows, due to the mode combination effects outlined in Figure 7. Third, there is the general reduction in cross-peak intensities in both calculated and measured spectra. Based on these observations, we conclude that there is evidence to suggest that Myc2345 forms unstacked G-quartets in the absence of ions, and we depict in Figure 9 how such a structure may look. In this model, on the addition of potassium ions, the G-quartets stack together to form a different structure presumed to be a G-quadruplex structure and based on the appearance of the two new coupling peaks, it is furthermore plausible that in such a structure, the guanine and/or thymine bases in the end-caps of the DNA sequence couple to the stacked quartets. Although solution NMR measurements of Myc2345 determined only a single DNA conformation,^26^ it is a possibility that the high concentration in our gel samples may promote the formation of other G4 conformations. However, assuming polymorphs consist of the same number of stacked G-quartets, it would be difficult to distinguish between these polymorphs due to the guanine carbonyl stretch frequency being the same. The abundance of a single strand versus a G4 is, however, more easily estimated, and an upper bound of 4% of single strand DNA has been estimated for the 100 mM K^+^ sample. Details surrounding this estimation can be seen in Figure S12.

## Conclusions

We have tracked the behavior of 102 distinct and identifiable vibrational couplings during the process of Myc2345 G-quadruplex folding, using EVV 2DIR spectroscopy, by titrating these features as a function of potassium ion concentration. The initial 102 peaks are modulated during folding in a number of ways, including changes in intensity and frequency shifts plus the formation of two new and additional coupling peaks. Assignments of spectral features and explanations for the cause of changes to spectral features were made with the aid of quantum-chemical (density functional theory) vibrational analysis and calculations of 2DIR spectra. Altogether, we find that there is evidence to suggest that Myc2345 forms unstacked G-quartet-resembling structures in the absence of ions and that a different structure, presumed to be a G-quadruplex, forms upon potassium ion addition. More generally, our work demonstrates that it is possible to obtain and interpret details about short-range couplings even in the absence of long-range order. Second, it shows that a high density of interpretable features can be obtained using EVV 2DIR spectroscopy with high fidelity.

## Supporting Information Available

Details on the data analysis procedure, potassium ion concentration dependence of intensities of additional peaks, structures for calculated EVV 2DIR spectra, the calculated spectra of an isolated guanine (PDF)
Vibrations of selected G4 modes (ZIP)
Gaussian input files (ZIP)

## Abbreviations

EVV 2DIR: electron-vibration-vibration two-dimensional infrared
G4: G-quadruplexes,
DFT: density functional theory
