# Supplementary material for: The Transition from Unfolded to Folded G-Quadruplex DNA Analyzed and Interpreted by Two-Dimensional Infrared Spectroscopy
Source: J Am Chem Soc. 2023 Aug 30;145(36):19622–32. doi: 10.1021/jacs.3c04044 (PMC10510320; doi:10.1021/jacs.3c04044)
Supplement: Supplementary file 1 — ja3c04044_si_001.pdf [file ja3c04044_si_001.pdf]

The Transition from Unfolded to Folded G-Quadruplex DNA Analysed and Interpreted by Two-Dimensional Infrared Spectroscopy

A. Larasati Soenarjo[1], Zhihao Lan[2], Igor V. Sazanovich[3], Yee San Chan[1], Magnus Ringholm[4], Ajay Jha[2,5], David R. Klug[1]*

Affiliations:

1. Department of Chemistry, Imperial College London, White City Campus, London W12 0BZ, United Kingdom
2. Rosalind Franklin Institute, Harwell Campus, Didcot, OX11 0QX, United Kingdom
3. Central Laser Facility, Research Complex at Harwell, STFC Rutherford Appleton Laboratory, Harwell, Oxford OX11 0QX, United Kingdom.
4. Hylleraas Centre for Quantum Molecular Sciences, Department of Chemistry, UiT The Arctic University of Norway, N-9037 Tromsø, Norway
5. Department of Pharmacology, University of Oxford, Oxford, OX1 3QT, United Kingdom

*Corresponding author. Email: d.klug@imperial.ac.uk

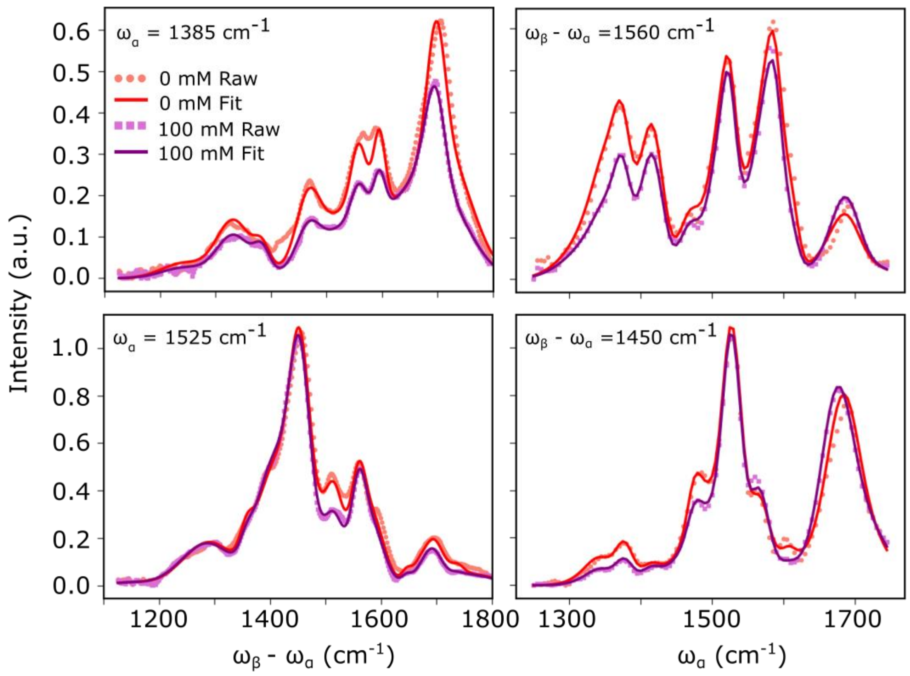

Figure S1. Comparison of raw and fitted experimental EVV 2DIR spectra of 1 mM Myc2345 in the presence of 0 and 100 mM K^+. Line cuts comparing raw (dotted line) and fitted (solid line) EVV 2DIR spectra of 1 mM Myc2345 with 0 K^+ (orange) and 100 mM K^+ (purple) at frequencies ω_α =1385 cm-1 (top left), ω_α =1525 cm-1 (bottom left), ω_β - ω_α =1560 cm-1 (top right) and ω_β - ω_α =1560 cm-1 (bottom right).

Table S1. Chi-squared values of the 2D Gaussian fitting of the experimental EVV 2DIR spectra of 1 mM Myc2345 in the presence of varying concentrations of potassium ions.

$[K^+]$ / mM	χ^2
0	16.8
5	7.7
10	4.6
100	2.9

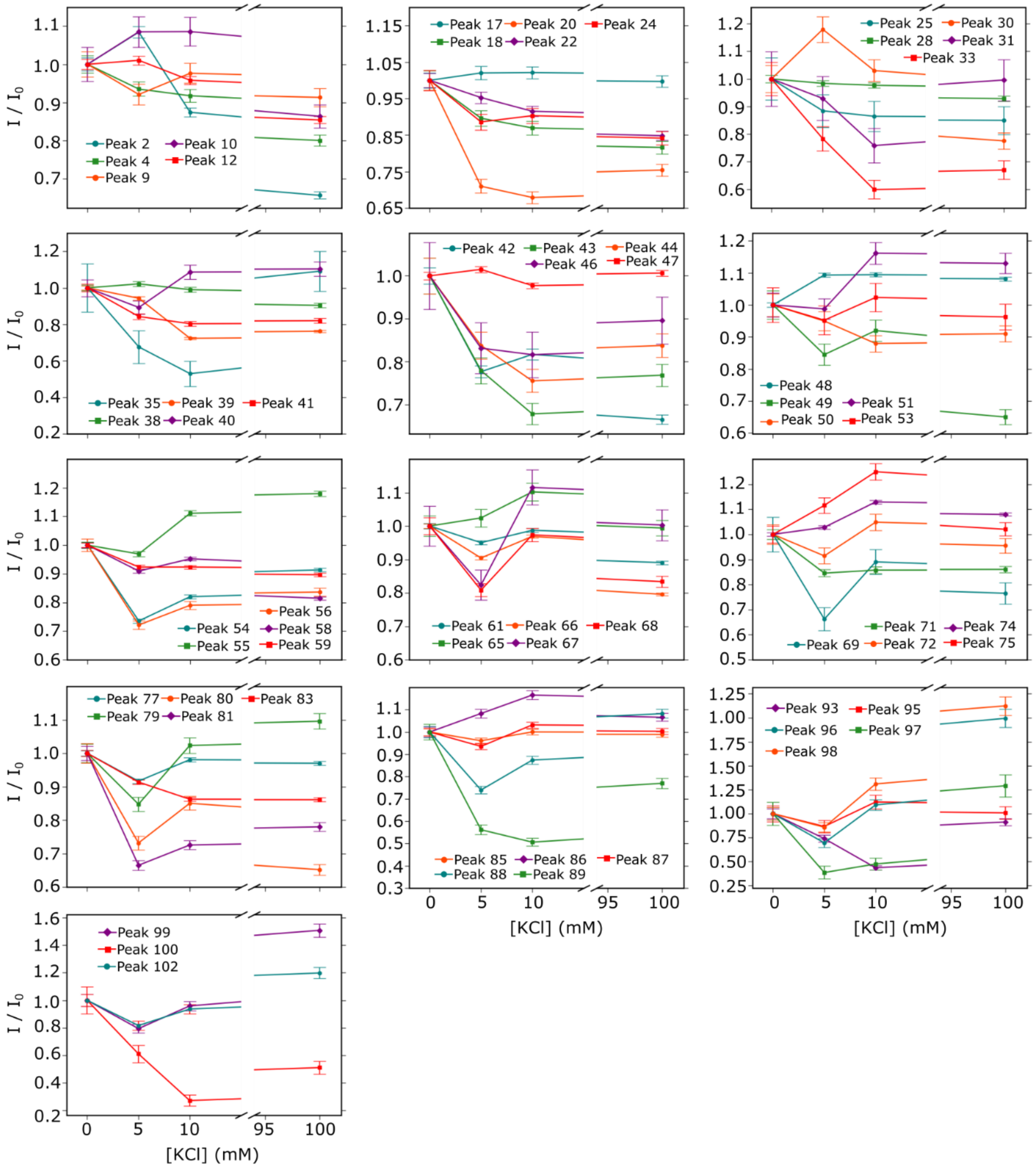

Figure S2. Relative intensity of cross-peaks as a function of potassium ion concentration. Intensity is presented as a fraction of the intensity in the absence of K^+ ions. The experimental EVV 2DIR spectra of Myc2345-K^+ were fitted with a total of 102 2D Gaussian functions. The intensity traces of cross-peaks observed to be increasing or decreasing with [K^+], and the intensity traces of new vibrational couplings can be found in the main manuscript in Figures 5 and 6b, respectively.

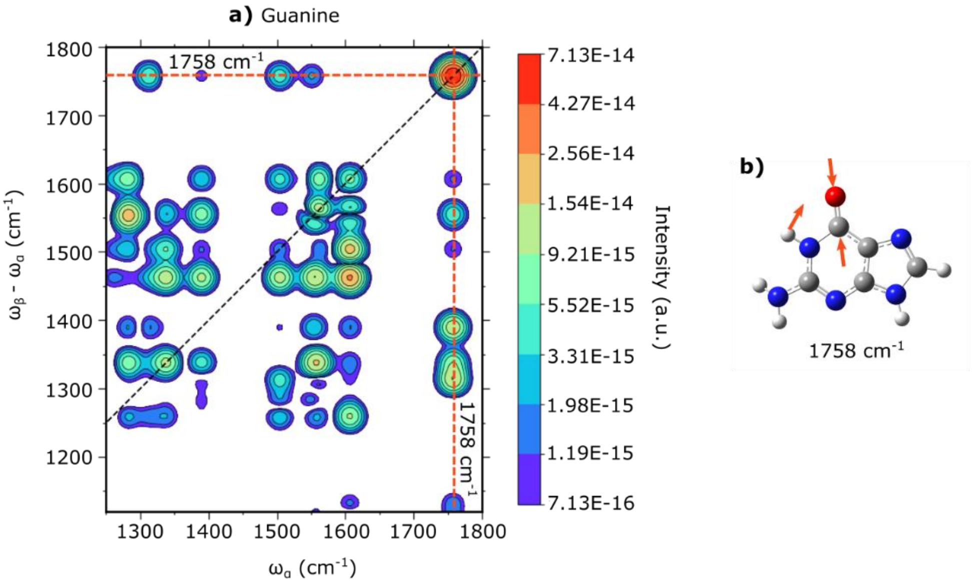

Figure S3. Computational EVV 2DIR spectrum of isolated guanine base. a, Computational EVV 2DIR spectrum of a guanine base. An isolated guanine base has one carbonyl stretch mode at 1758 cm^{-1}, which gives rise to a row and column of cross-peaks. **b,** Depiction of guanine carbonyl stretch mode in an isolated guanine base.

		1 5 10 15 20 25
**a)** Pu27		TGGGGAGGGTGGGGAGGGTGGGGAAGG
		5 10 15 20 25
**b)** Myc2345		TGAGGGTGGGGAGGGTGGGGAA
**c)** Myc2345-WT		TGAGGGTGGGTAGGGTGGGGAA
**d)** MycL1		TGAGGGTGGGTAGGGTGGGTAA

Figure S4. Sequence comparison of the G4-forming sequence in the human c-myc promoter region from different publications. a, Pu27, the 27-nt purine-rich wild type sequence found in the NHE of the c-myc promoter. **b,** Myc2345 sequence used for the EVV 2DIR measurements in this work. **c,** Wild type Myc2345 sequence as used in NMR structure PDB:7KBV.[1] **d,** MycL1 – altered Myc2345 sequence used in CD measurements in Ref.[2] Bases in red signify those that differ from the Myc2345 sequence used in the EVV 2DIR measurements.

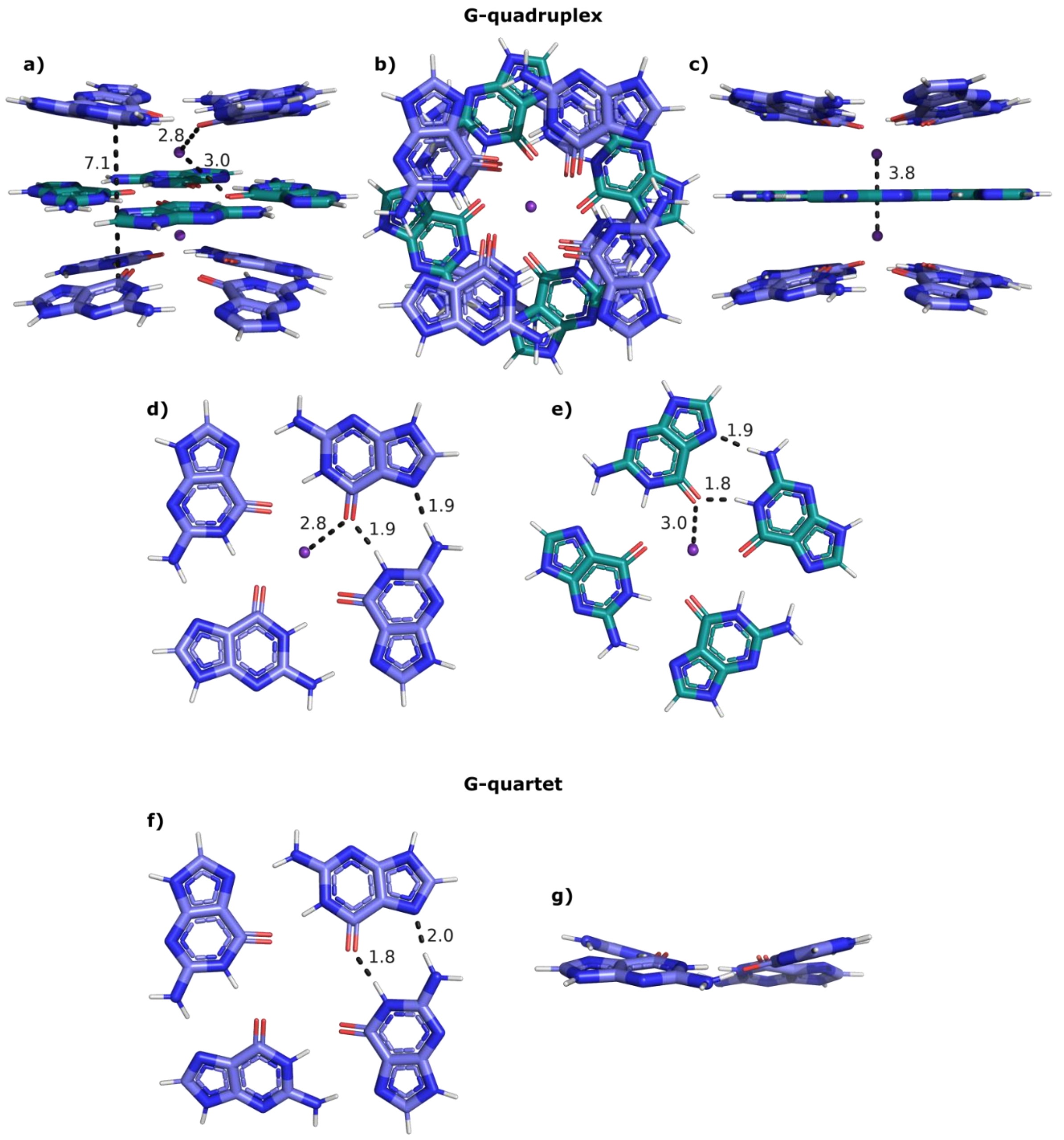

Figure S5. The G-quadruplex and G-quartet structure used for the calculation of the computational EVV 2DIR spectrum. a-e, Structure of the G-quadruplex. Figure **(b)** shows a top-down view of the G-quadruplex. The terminal quartets (purple) are aligned with each other, and the central quartet (green) is ~45° rotated with respect to the terminal quartets. Figures **(d)** and **(e)** presents the terminal and central quartets of the G-quadruplex, respectively. **f-g,** Structure of the G-quartet from **(f)** top-down view and **(g)** side view.

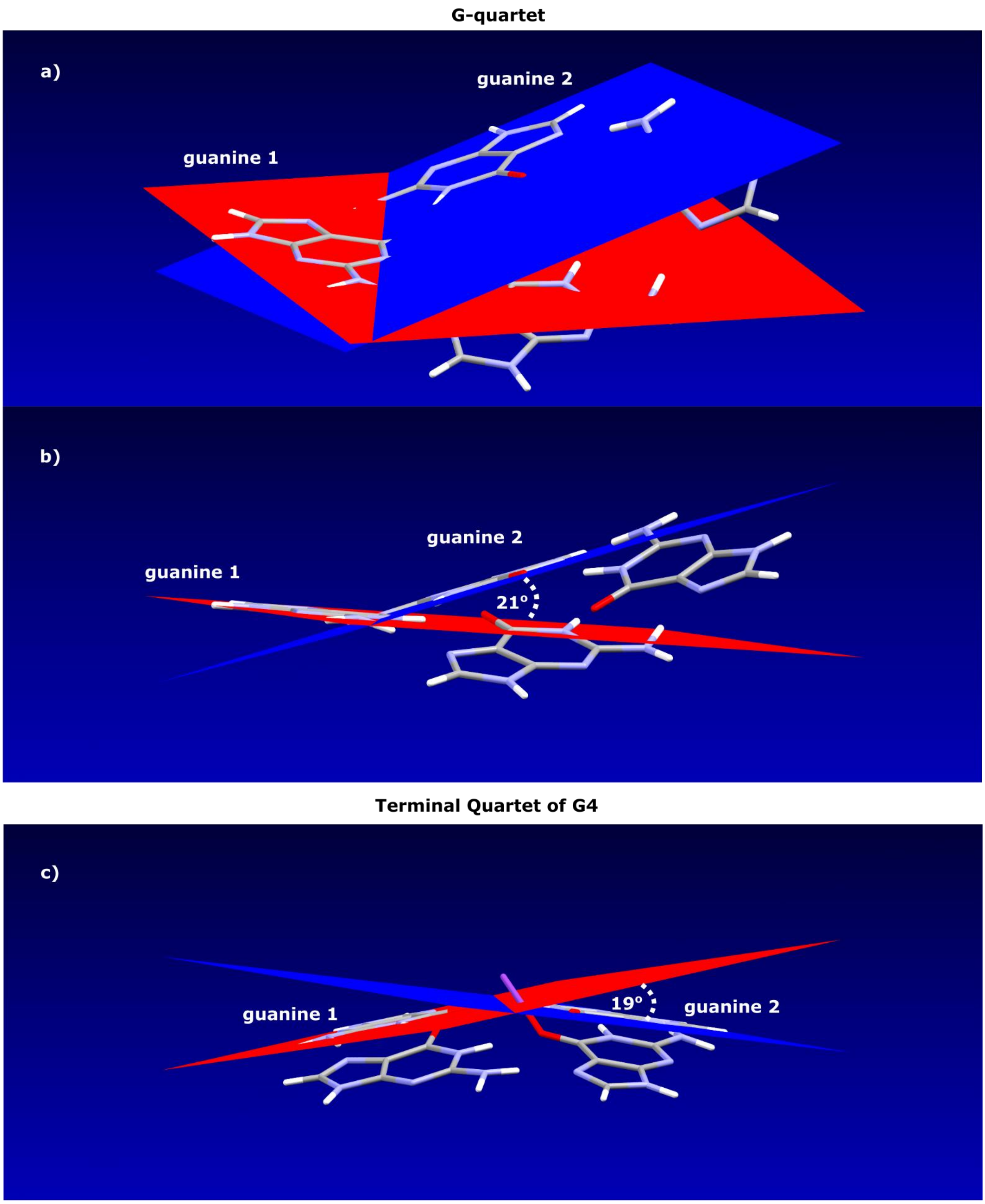

Figure S6. Comparison of structures of G-quartet and terminal quartets in a G4. Structure of **(a-b)** G-quartet and **(c)** a terminal quartet of G4, showing the intersection of molecular planes of adjacent guanine bases. The guanine molecular planes intersect at an angle of 21° in the G-quartet and 19° in the terminal quartets of the G4.

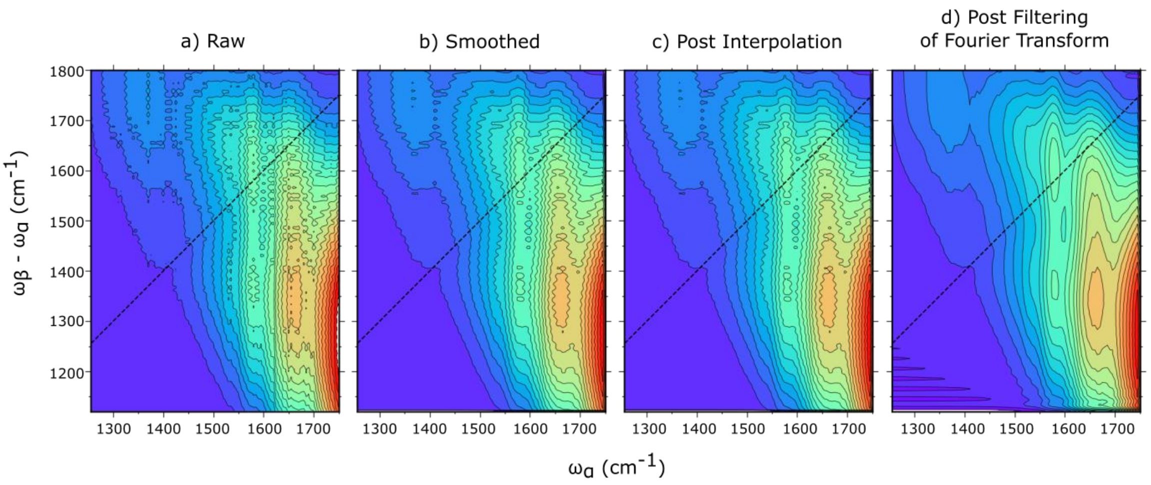

Figure S7. Processing of EVV 2DIR spectrum of glass coverslip used to normalise EVV 2DIR spectra of Myc2345. EVV 2DIR spectrum of glass coverslip **(a)** raw, **(b)** after smoothing by averaging over nearest neighbours, **(c)** after interpolation along the vertical axis, **(d)** after applying a low-pass filter to the Fourier transform.

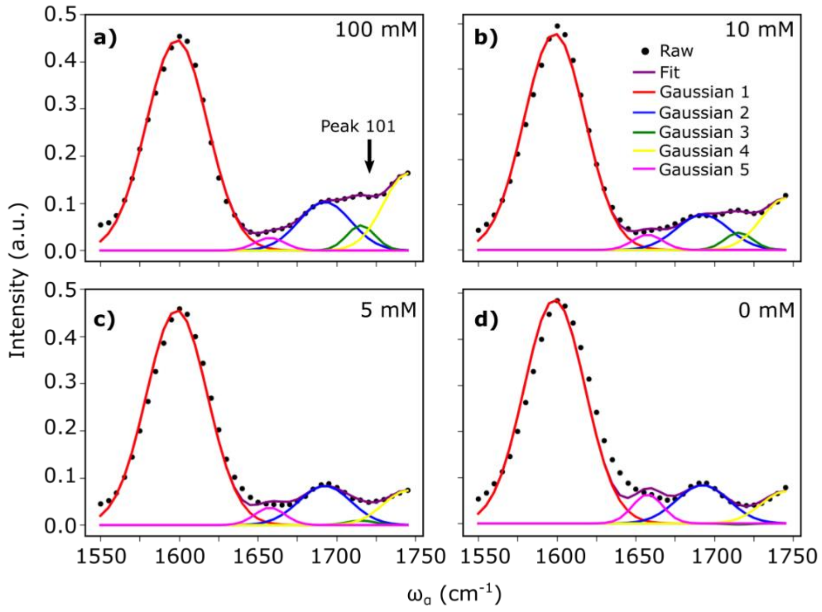

Figure S8. a-b, Fitting of line cuts of experimental EVV 2DIR spectra of 1 mM Myc2345 in the presence of 0, 5, 10 and 100 mM K^+ at ω_β - ω_α = 1675 cm-1 with a sum of 1D Gaussians. The 100 mM K^+ line cut (a) was first fitted with five 1D gaussians with no parameter boundaries. The 10, 5 and 0 mM K^+ line cuts (b-d) were subsequently fitted with five 1D Gaussians, with the widths and locations fixed to the values determined from the fit of the 100 mM K^+ line cut and no boundaries for the intensities. These fits show peak 101 (green Gaussian feature) growing in as [K^+] is increased.

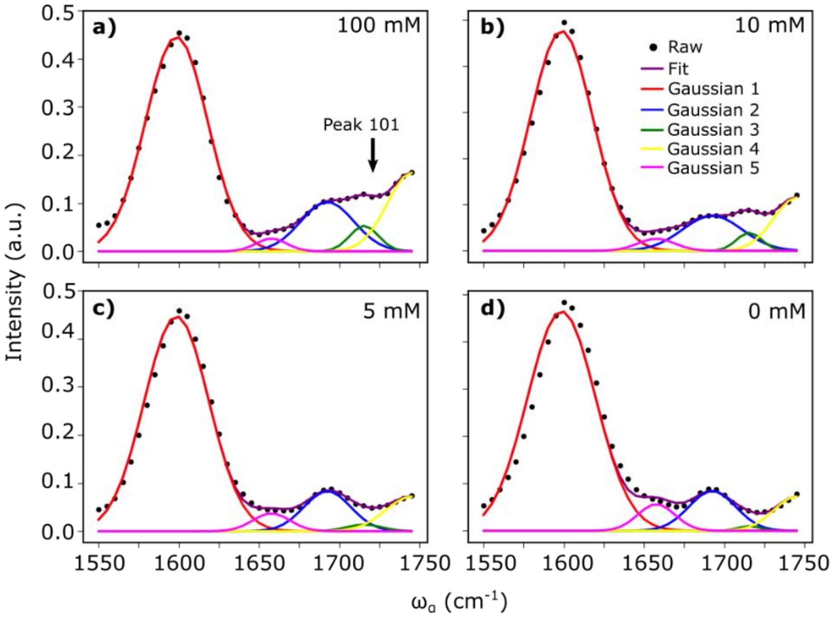

Figure S9. a-d, Fitting of line cuts of experimental EVV 2DIR spectra of 1 mM Myc2345 in the presence of 0, 5, 10 and 100 mM K^+ at ω_β - ω_α = 1675 cm-1 with a sum of 1D Gaussians with unconstrained widths. The 100 mM K^+ line cut (a) was first fitted with five 1D gaussians with no parameter boundaries. The 10, 5 and 0 mM K^+ line cuts (b-d) were subsequently fitted with five 1D Gaussians. The locations were fixed to the values determined from the fit of the 100 mM K^+ line cut. The widths of the gaussians were allowed to vary ±2 cm^{-1} from the value determined in the fit of the 100 mM K^+ line cut. No boundaries were placed for the intensities of the gaussians. These fits show peak 101 (green Gaussian feature) growing in as [K^+] is increased, and that it is not an artifact of constraining the widths of Gaussians.

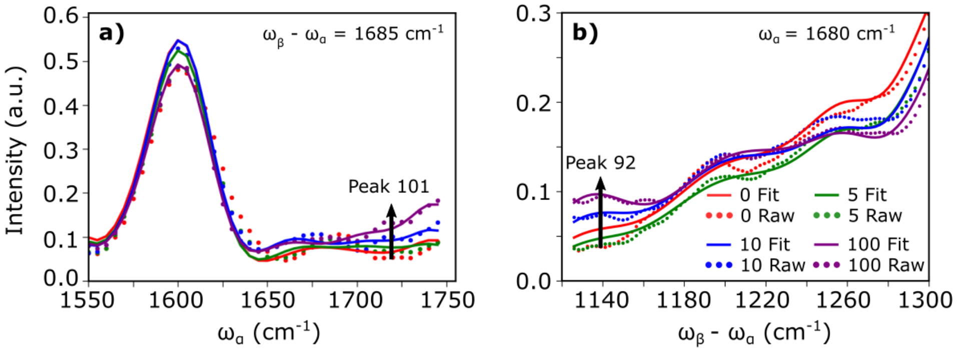

Figure S10. A comparison of raw and fitted data of 1 mM Myc2345 in the presence of 0, 5, 10 and 100 mM K^+ in the regions where new peaks 101 (a) and 92 (b) are observed to appear on addition of K^+ ions. The fitted data shown here is from the fitting of the entire 2D spectra with a sum of 102 2D Gaussians, and is not to be confused with fitting of line cuts with a sum of 1D Gaussians.

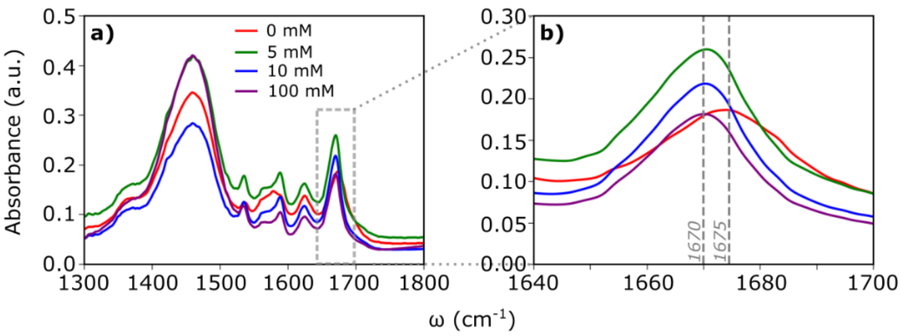

Figure S11. FTIR spectra of solutions of 1 mM Myc2345 in the presence of 0, 5, 10 and 100 mM K^+, prepared as described in Materials and Methods excluding gel formation. The guanine carbonyl stretch feature is observed to red-shift from 1675 cm^{-1} (0 mM K^+) to 1670 cm^{-1} (100mM K^+).

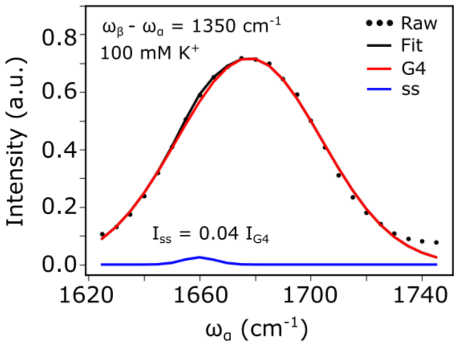

Figure S12. The upper limit for the abundance of single stranded DNA can be estimated due to the difference in guanine carbonyl stretch frequencies between single strand and complexed guanine. The calculated EVV 2DIR spectrum of an isolated guanine shown in Figure S3a in the Supplementary Information suggests that the carbonyl stretch of single strand guanine should give rise to a column of cross-peaks with comparable intensities to the G4 GCO II column in Figure 7b in the manuscript. As the 2D fits of the EVV 2DIR spectra of Myc2345 did not require a column of gaussians between ω_α = 1660-1673 cm^{-1} to take account of the presence of single strand guanine, it suggests the G4 structure dominates. One possible approach towards estimating the abundance of single-strand versus G4 DNA would be to fit a horizontal line cut in the guanine carbonyl stretch region with a peak between ω_α = 1660-1673 cm^{-1} for the single strand guanine in addition to the G4 guanine peak. Figure S12 shows a horizontal line cut of the 100 mM K^+ EVV 2DIR spectrum at $\omega_\beta - \omega_\alpha$ = 1350 cm^{-1} fitted with a 1D gaussian at ω_α = 1678 cm^{-1} (G4) and another at ω_α = 1660 cm^{-1} (single strand). The maximum intensity of the single strand gaussian is 0.04 times the intensity of the G4 gaussian. Whilst acknowledging the limitations of the extent and the suitability of the underlying data for this analysis, and assuming the cross-sections of these two couplings are equal, an estimate stemming from it suggests an upper bound of 4% of single strand DNA in the 100 mM K^+ sample.

References

1. Dickerhoff, J., Dai, J. & Yang, D. Structural recognition of the MYC promoter G-quadruplex by a quinoline derivative: insights into molecular targeting of parallel G-quadruplexes. *Nucleic Acids Res* **49**, 5905–5915 (2021).
2. Friedman, S. J. & Terentis, A. C. Analysis of G-quadruplex conformations using Raman and polarized Raman spectroscopy. *Journal of Raman Spectroscopy* **47**, 259–268 (2016).
