# Supplementary figures and images for: The Transition from Unfolded to Folded G-Quadruplex DNA Analyzed and Interpreted by Two-Dimensional Infrared Spectroscopy
Source: J Am Chem Soc. 2023 Aug 30;145(36):19622–32. doi: 10.1021/jacs.3c04044 (PMC10510320; doi:10.1021/jacs.3c04044)

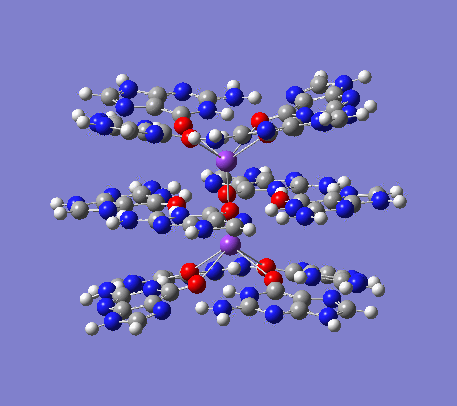

Supplement: Supplementary file 2 — ja3c04044_si_002.zip [file ja3c04044_si_002.zip › G4 vibrations/10_G4_mode365_1132cm-1_side_view.gif]

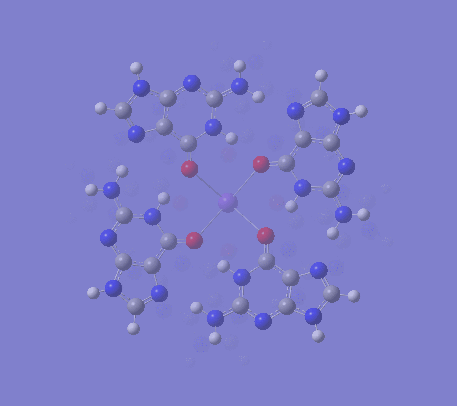

Supplement: Supplementary file 2 — ja3c04044_si_002.zip [file ja3c04044_si_002.zip › G4 vibrations/11_G4_mode366_1132cm-1_birds_eye_view.gif]

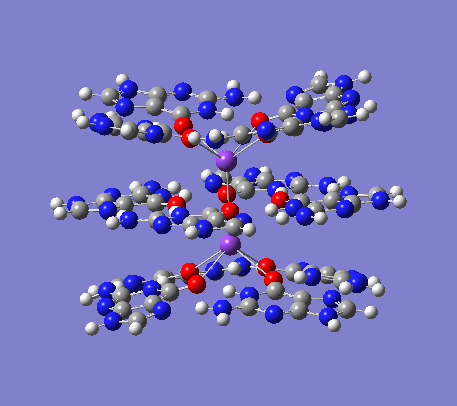

Supplement: Supplementary file 2 — ja3c04044_si_002.zip [file ja3c04044_si_002.zip › G4 vibrations/12_G4_mode366_1132cm-1_side_view.gif]

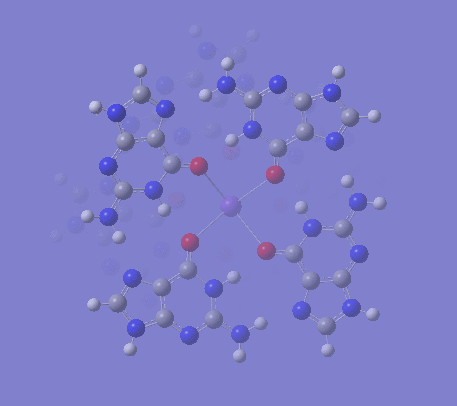

Supplement: Supplementary file 2 — ja3c04044_si_002.zip [file ja3c04044_si_002.zip › G4 vibrations/13_G4_mode367_1132cm-1_birds_eye_view.gif]

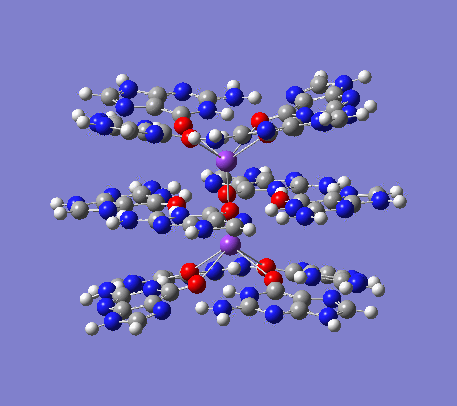

Supplement: Supplementary file 2 — ja3c04044_si_002.zip [file ja3c04044_si_002.zip › G4 vibrations/14_G4_mode367_1132cm-1_side_view.gif]

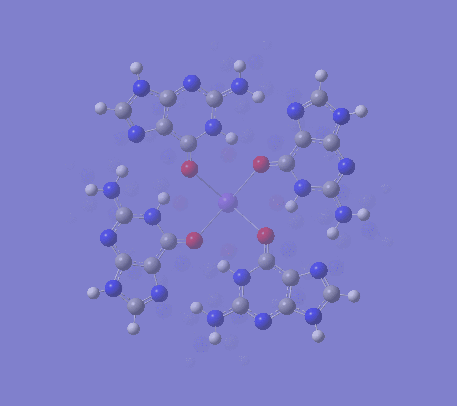

Supplement: Supplementary file 2 — ja3c04044_si_002.zip [file ja3c04044_si_002.zip › G4 vibrations/15_G4_mode513_1675cm-1_birds_eye_view.gif]

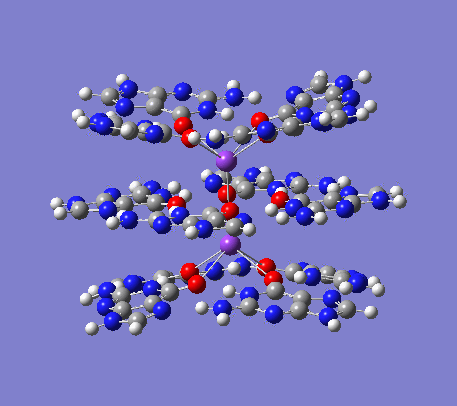

Supplement: Supplementary file 2 — ja3c04044_si_002.zip [file ja3c04044_si_002.zip › G4 vibrations/16_G4_mode513_1675cm-1_side_view.gif]

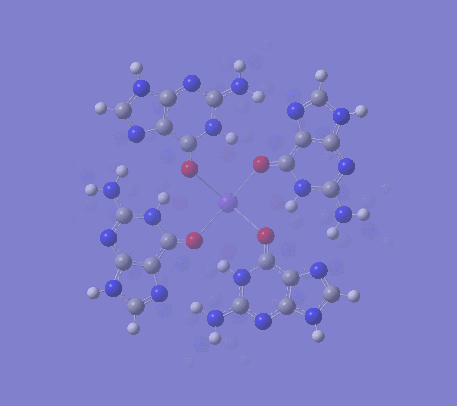

Supplement: Supplementary file 2 — ja3c04044_si_002.zip [file ja3c04044_si_002.zip › G4 vibrations/1_G4_mode514_1679cm-1_birds_eye_view.gif]

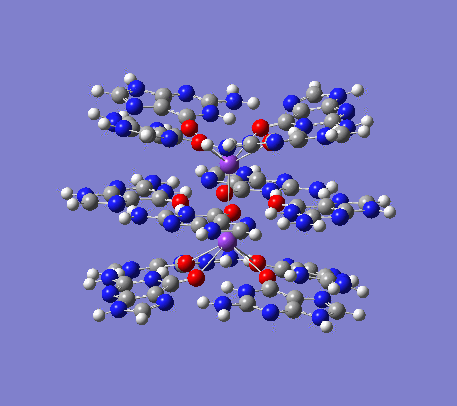

Supplement: Supplementary file 2 — ja3c04044_si_002.zip [file ja3c04044_si_002.zip › G4 vibrations/2_G4_mode514_1679cm-1_side_view.gif]

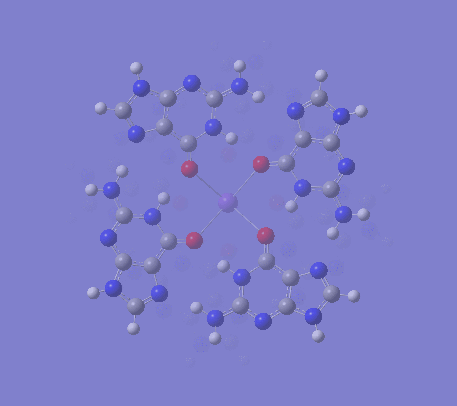

Supplement: Supplementary file 2 — ja3c04044_si_002.zip [file ja3c04044_si_002.zip › G4 vibrations/3_G4_mode515_1679cm-1_birds_eye_view.gif]

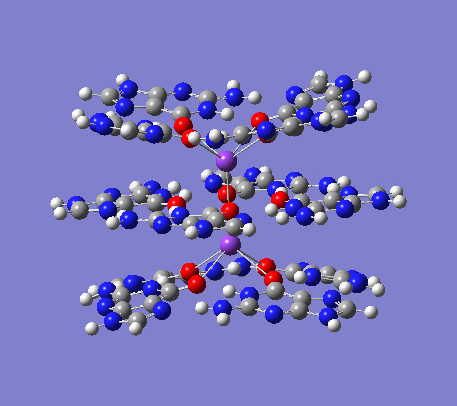

Supplement: Supplementary file 2 — ja3c04044_si_002.zip [file ja3c04044_si_002.zip › G4 vibrations/4_G4_mode515_1679cm-1_side_view.gif]

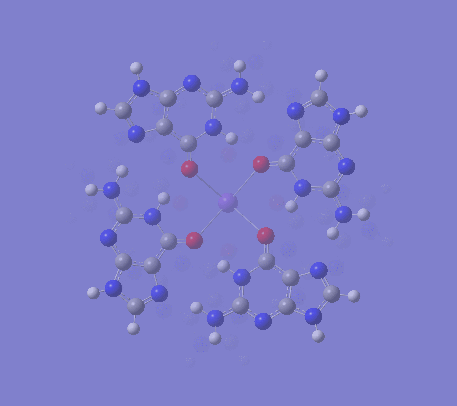

Supplement: Supplementary file 2 — ja3c04044_si_002.zip [file ja3c04044_si_002.zip › G4 vibrations/5_G4_mode516_1692cm-1_birds_eye_view.gif]

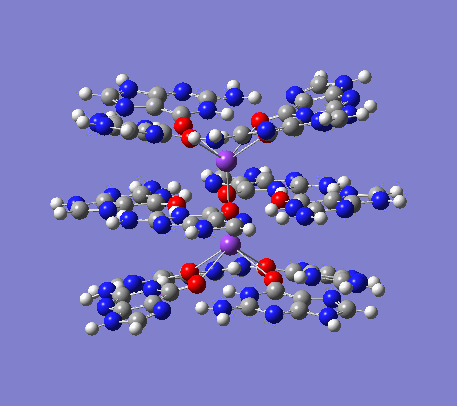

Supplement: Supplementary file 2 — ja3c04044_si_002.zip [file ja3c04044_si_002.zip › G4 vibrations/6_G4_mode516_1692cm-1_side_view.gif]

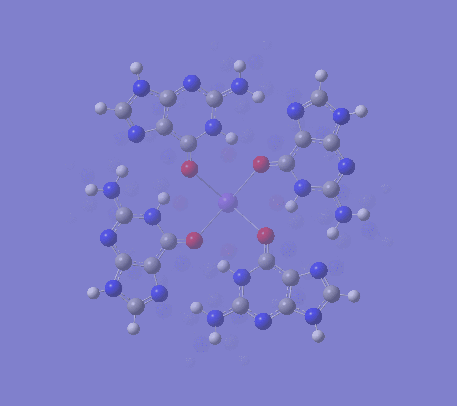

Supplement: Supplementary file 2 — ja3c04044_si_002.zip [file ja3c04044_si_002.zip › G4 vibrations/7_G4_mode364_1132cm-1_birds_eye_view.gif]

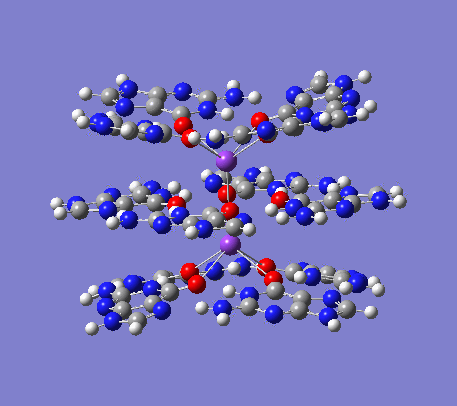

Supplement: Supplementary file 2 — ja3c04044_si_002.zip [file ja3c04044_si_002.zip › G4 vibrations/8_G4_mode364_1132cm-1_side_view.gif]

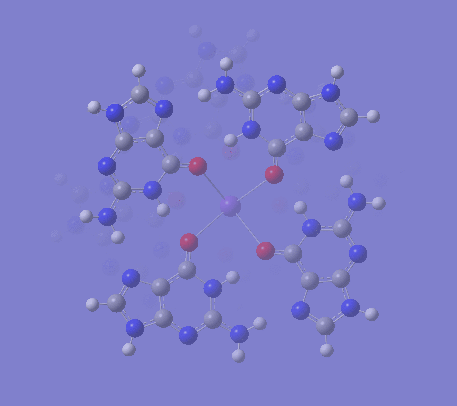

Supplement: Supplementary file 2 — ja3c04044_si_002.zip [file ja3c04044_si_002.zip › G4 vibrations/9_G4_mode365_1132cm-1_birds_eye_view.gif]
